# Polyanhydride‐Based Microparticles for Programmable Pulsatile Release of Diphtheria Toxoid (DT) for Single‐Injection Self‐Boosting Vaccines

**DOI:** 10.1002/adma.202501168

**Published:** 2025-05-15

**Authors:** Linzixuan Zhang, Ruiqing Xiao, Wenhao Gao, Johnny Garcia, Xinyan Pan, John L. Daristotle, Timothy Forster, Jooli Han, Mehr Chaddah, Dhruv Varshney, Nandita Menon, Kevin J. McHugh, Benjamin J. Pedretti, Jing Ying Yeo, Xin Yang, Sydney MacDonald, Robert Langer, Ana Jaklenec

**Affiliations:** ^1^ Department of Chemical Engineering Massachusetts Institute of Technology Cambridge MA 02139 USA; ^2^ David H. Koch Institute for Integrative Cancer Research Massachusetts Institute of Technology Cambridge MA 02139 USA; ^3^ Department of Biological Engineering Massachusetts Institute of Technology Cambridge MA 02139 USA; ^4^ Department of Bioengineering Rice University Houston TX 77005 USA; ^5^ Department of Chemistry Rice University Houston TX 77005 USA; ^6^ Department of Biomedical Engineering Seoul National University College of Medicine Seoul 03080 Republic of Korea

**Keywords:** antigen stability, microparticles, protein encapsulation, release kinetics, single‐administration vaccines

## Abstract

Vaccination remains a critical tool in preventing infectious diseases, yet its effectiveness is undermined by under‐immunization, particularly for vaccines requiring multiple doses that patients fail to complete. To address this challenge, the development of single‐injection platforms delivering self‐boosting vaccines has gained significant attention. Despite some advances, translating these platforms into clinical applications has been limited. In this study, a novel polyanhydride‐based polymeric delivery platform is introduced, designed for single‐injection self‐boosting vaccines, replacing multiple doses. Over 20 polyanhydride polymers are synthesized and screened, ultimately down selecting to 6 for in vitro studies, and 2 for in vivo studies. Using diphtheria toxoid (DT) as a model antigen, programmed pulsatile release with a narrow window is demonstrated, ideal for self‐boosting immunization. The platform effectively protects the pH‐sensitive antigen before release, achieving recovery rate of 39.7% to 89.7%. The system's tunability is further enhanced by machine learning algorithms, which accurately predict release profiles, confirmed through experimental validation. In vivo studies in a mouse model reveals that the platform induces DT‐specific antibody responses comparable to those generated by traditional multi‐dose regimens. Collectively, these findings highlight the potential of this platform to deliver various vaccines, offering a potentially promising solution to the global challenge of under‐immunization.

## Graphical abstract

This study presents a polyanhydride‐based delivery platform for single‐injection, self‐boosting vaccines. The system enables programmable, pulsatile release of diphtheria toxoid with high antigen recovery and is guided by machine learning for tunability. In vivo results show immune responses comparable to multi‐dose regimens, highlighting its potential to improve vaccine accessibility and reduce under‐immunization.

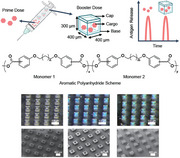

## 1 Introduction

Vaccination remains the most efficient and cost‐effective strategy for preventing infectious diseases, saving an estimated 6 million lives annually.^[^ ^1^, ^2^ ^]^ The introduction of vaccines has dramatically reduced the incidence of vaccine‐preventable diseases such as diphtheria, measles, and mumps. The COVID‐19 pandemic further highlighted their critical role, with full vaccination reducing severe acute respiratory syndrome coronavirus 2 (SARS‐CoV‐2) infection rates by 85–95%, significantly decreasing morbidity and mortality.^[^ ^1^, ^3^ ^]^ Despite these advancements, under‐immunization remains a major global health issue, particularly in developing countries where healthcare access is limited.^[^ ^4^ ^]^ A major cause of under‐immunization is the requirement for multiple vaccine doses, with nearly 50% of infants in developing regions failing to complete multi‐dose regimens for vaccines that prevent diseases such as tetanus, diphtheria, and pertussis (TDaP).^[^ ^5^, ^6^ ^]^ Addressing this issue through healthcare infrastructure improvements across various countries has been challenging, making it essential to explore technical solutions that can be implemented within existing systems.^[^ ^7^, ^8^ ^]^

Single‐injection platforms, designed to consolidate multiple vaccine doses into one administration, offer a possible solution to improve vaccine completion rates.^[^ ^9^, ^10^, ^11^ ^]^ These platforms enable the delivery of self‐boosting vaccines, which mimic traditional multi‐dose schedules by providing long‐term, intermittent antigen exposure. The development of such platforms has primarily focused on synthetic polymers, such as poly(lactic‐*co*‐glycolic acid) (PLGA) and polyanhydrides, which can offer controlled release.^[^ ^12^, ^13^, ^14^, ^15^ ^]^ However, most existing systems rely on continuous release kinetics, which are not optimal for self‐boosting vaccines.^[^ ^11^ ^]^ Instead, pulsatile release kinetics – delivering antigen doses at preset intervals – is ideal as it closely mirrors traditional vaccination schedules.^[^ ^16^ ^]^ This resemblance may facilitate regulatory approval and address concerns about safety and efficacy.^[^ ^11^, ^17^ ^]^

A significant hurdle in advancing single‐injection vaccine platforms is the reliance on traditional fabrication methods that incorporate organic solvents like dichloromethane.^[^ ^11^, ^18^, ^19^, ^20^ ^]^ These solvents not only pose safety concerns but also jeopardize the long‐term stability of encapsulated antigens.^[^ ^19^, ^21^ ^]^ To address these challenges, innovative solvent‐free fabrication techniques have been developed.^[^ ^22^, ^23^, ^24^ ^]^ These methods enable the production of fillable core‐shell microparticles (MPs) that aim precise cargo release through controlled polymer composition and structural design.

Although PLGA is widely used for MP shells due to its FDA‐approved status and customizable degradation profiles, its degradation products—lactic and glycolic acid—create an acidic microenvironment that can destabilize sensitive antigens.^[^ ^25^, ^26^, ^27^, ^28^ ^]^ The fact that PLGA is a bulk erosion polymer further exacerbates this issue by exposing the encapsulated antigen to an acidic environment throughout the degradation process. Efforts to mitigate this issue by incorporating basic excipients have shown only partial success, as these approaches often accelerate antigen release prematurely.^[^ ^29^ ^]^ This limitation underscores the urgent need for alternative polymeric materials that provide a more stable environment while ensuring controlled antigen release tailored for single‐injection vaccine systems.

To address these limitations, in this study, we synthesized new polyanhydrides and fabricated a new class of microparticles that can release vaccines in pulses. Polyanhydride is one of the most well‐studied polymer materials for drug delivery applications, with extensive research dedicated to its synthesis, degradation kinetics, biocompatibility, and chemical reactivity.^[^ ^30^, ^31^ ^]^ Polyanhydrides have also been used in several clinically approved medical products, such as Gliadel.^[^ ^32^ ^]^ In this work, polyanhydride was chosen due to its potential to address the challenges associated with PLGA.^[^ ^30^ ^]^ One key advantage of polyanhydride is its ability to achieve surface erosion by tuning monomer compositions, thereby providing better protection to hydrolytically‐sensitive antigens by only exposing them to significant amounts of water at the preset release time point.^[^ ^33^, ^34^, ^35^ ^]^ Specifically, the exterior surface of the polyanhydride‐based MPs degrade gradually into oligomers and monomers after injection, while the core remains relatively dry and maintains a stable pH, creating an environment conducive to preserving sensitive antigens until the release point is reached.^[^ ^36^ ^]^ This feature minimizes pH reduction in the particle core, preserving the stability of pH‐sensitive antigens. Additionally, the structural diversity of polyanhydride monomers can be leveraged to adjust degradation byproduct solubility, which may help mitigate pH‐related issues and maintain an environment favorable for antigen stability.^[^ ^37^, ^38^, ^39^ ^]^

Given that single‐injection platforms for self‐boosting vaccines need to simulate traditional multi‐dose administration schedules, which can span months or even years, aromatic polyanhydrides were deemed appropriate for this application.^[^ ^40^ ^]^ Unlike aliphatic polyanhydrides, which typically degrade within days, aromatic polyanhydrides can maintain their mechanical structure for months to years, making them more suitable for long‐term vaccine delivery.^[^ ^37^ ^]^ Moreover, the hydrophobic nature of aromatic groups helps to further limit the aqueous solubility of degradation byproducts, thereby maintaining a more stable core environment.^[^ ^38^ ^]^ Experimental tests indicated that the pH of saturated aqueous solutions of aromatic polyanhydride degradation byproducts was less acidic compared to those of PLGA. This finding suggests that aromatic polyanhydrides could help maintain antigen stability, though variations in formulation may influence these outcomes.^[^ ^30^ ^]^

We selected DT as a model vaccine to demonstrate the effectiveness of the polyanhydride‐based core‐shell MP platform due to its pH sensitivity. Diphtheria toxoid, the detoxified form of diphtheria toxin, generates toxin‐neutralizing antibodies against diphtheria, an infectious disease caused by *Corynebacterium diphtheriae*.^[^ ^41^, ^42^ ^]^ Currently, the most common DT vaccine is administered as part of the alum‐adsorbed diphtheria‐tetanus‐pertussis vaccine, which requires multiple injections.^[^ ^43^ ^]^ PLGA‐based microspheres encapsulating DT for self‐boosting immunization have been previously reported, but their clinical application has been limited by stability challenges, including interactions with organic solvents during fabrication and the acidic environment generated by PLGA degradation.^[^ ^11^, ^44^ ^]^ DT serves as an ideal model for this platform due to its high thermal stability yet strong sensitivity to acidic conditions.^[^ ^45^ ^]^ It has been shown that DT remains stable at body temperature, with minimal loss of potency when stored at 37 °C for 2 to 6 months.^[^ ^46^ ^]^ However, DT rapidly loses activity in acidic environments.^[^ ^45^, ^47^ ^]^ By using DT, this study aims to demonstrate the platform's capability to protect acid‐sensitive antigens and deliver them effectively in a single‐injection, self‐boosting vaccine format.

In this study, we developed a polyanhydride‐based core‐shell MP platform for single‐injection, self‐boosting vaccines. We synthesized, screened, and selected suitable polyanhydride materials, optimized fabrication, and achieved programmable, pulsatile antigen release in vitro. Machine learning algorithms were employed to further refine the fabrication process. In vivo studies with the DT vaccine demonstrated that the platform effectively elicited immune responses comparable to traditional multi‐dose vaccines, showcasing its potential as an effective solution for addressing under‐immunization.

## 2 Results and Discussions

### 2.1 Design of Polyanhydride Core‐Shell MPs for Self‐Boosting Vaccines

**Figure 1:**
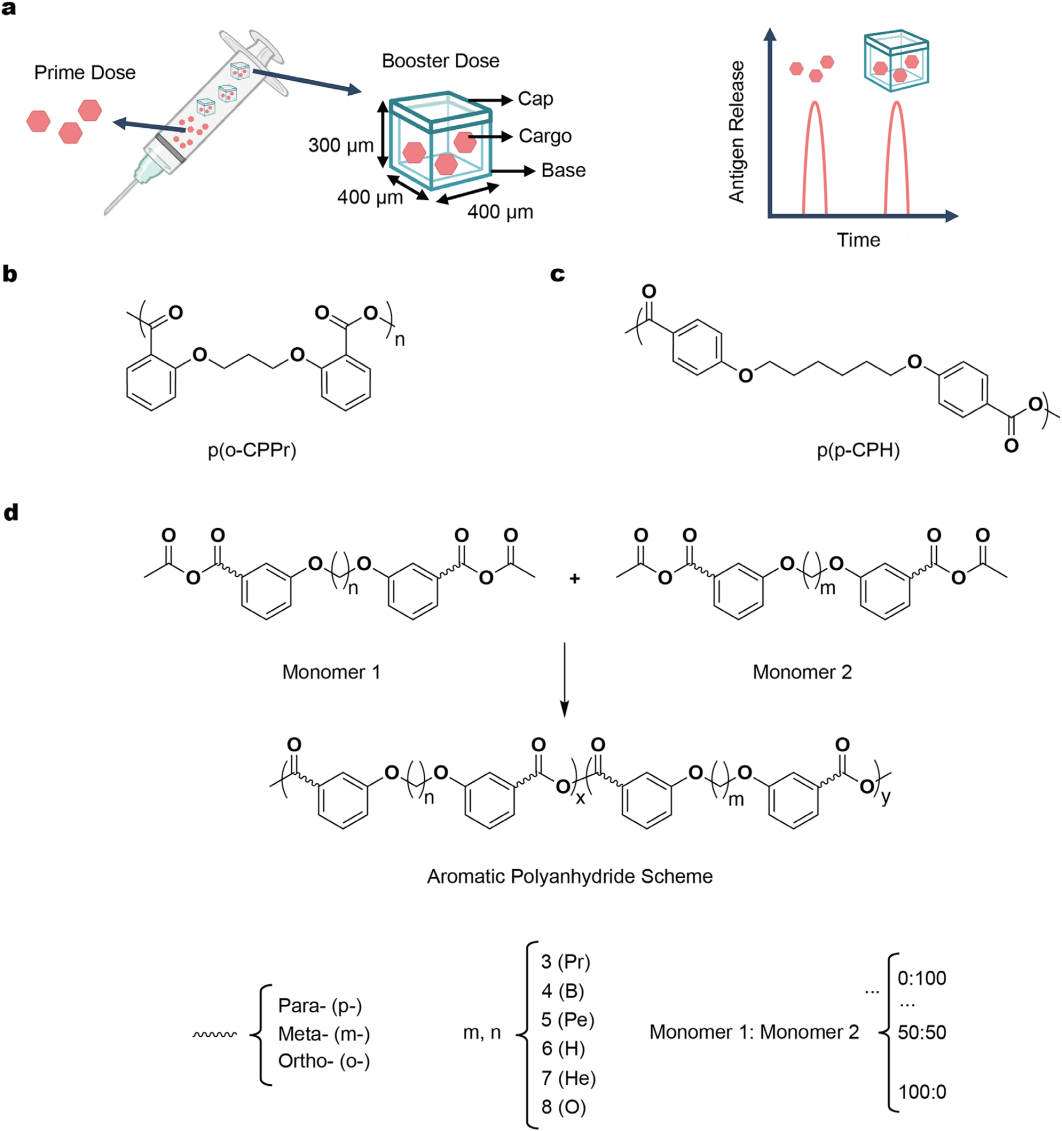
Design and material selection for polyanhydride core‐shell MPs. a) A single‐injection vaccine platform is designed to deliver a prime bolus dose along with a group of SEAL core‐shell MPs, where the bolus dose is administered immediately, and the booster dose is released at a predetermined time. Each SEAL core‐shell MP consists of three main components: a cap, a base, and encapsulated cargo. b,c) Chemical structures of two well‐studied aromatic polyanhydride polymers, p(o‐CPPr) (b) and p(p‐CPH) (c). d) An aromatic polyanhydride library was created by diversifying hydroxybenzoic acid derivatives and dibromides via synthesis routes, with additional variation in monomer feed ratios, as shown in the generalized structure scheme.

Most self‐boosting vaccine development has focused on polymer matrixes by encapsulating antigens in polymer‐based micro‐ or nanostructures.^[^ ^48^, ^49^ ^]^ Upon injection, the antigen is released according to predetermined kinetics, triggered by polymer biodegradation. In this study, we utilized a microfabrication method known as StampEd Assembly of polymer Layers (SEAL) to produce fillable core‐shell MPs.^[^ ^22^, ^50^ ^]^ Using the SEAL platform, a single‐injection vaccine with pulsatile release kinetics can be achieved by administering a soluble prime dose along with core‐shell MPs that release the drug at predetermined intervals (**Figure** **1**a). The design of SEAL‐based core‐shell MPs consists of a base that encapsulates the drug of interest and a cap for sealing. Each MP has dimensions of 400 µm × 400 µm × 300 µm (length, width, height), with an internal cavity measuring 300 µm × 300 µm × 200 µm (Figure 1a; Figure S1, Supporting Information). This design allows for a loading capacity of 18 nanoliters (nL) per MP, a significant increase from the original design by McHugh et al. with a capacity of only 1 nL (Figure S1, Supporting Information).^[^ ^22^ ^]^ The thinner walls of the cube‐shaped MPs used in this design, however, necessitate stricter mechanical strength requirements for the materials used in fabrication. Compared to traditional emulsion‐based MPs, the SEAL‐based fabrication of core‐shell MPs eliminates the need for toxic organic solvents and provides a high degree of tunability in the types, concentrations, and ratios of excipients. These parameters can be adjusted by simply modifying the filling solutions without requiring reformulation or changes to the fabrication process.

We developed a polyanhydride polymer library by expanding on two common aromatic polyanhydride compositions: poly(1,3‐bis(carboxyphenoxy)propane) (pCPP) and poly(1,6‐bis(carboxyphenoxy)hexane) (pCPH) (Figure 1b,c).^[^ ^51^ ^]^ The synthesis of these aromatic polyanhydrides involved three main steps: (1) alkylation of a hydroxybenzoic acid with a dibromide to form a diacid, (2) acetylation of the diacid to yield a prepolymer/monomer, and (3) melt condensation polymerization to produce the final polymer (Figure S2, Supporting Information).^[^ ^52^ ^]^ To expand the polymer library, we varied the synthesis by using different hydroxybenzoic acids and dibromides (Figure 1d). Additionally, adjusting the monomer feed ratios further diversified the library (Figure 1d). This approach yielded a wide variety of potential compositions, from which we selected over 20 representative examples and evaluated their compatibility with the MP fabrication process.

### 2.2 Screening and Fabrication of Polyanhydride Core‐Shell MPs

**Figure 2:**
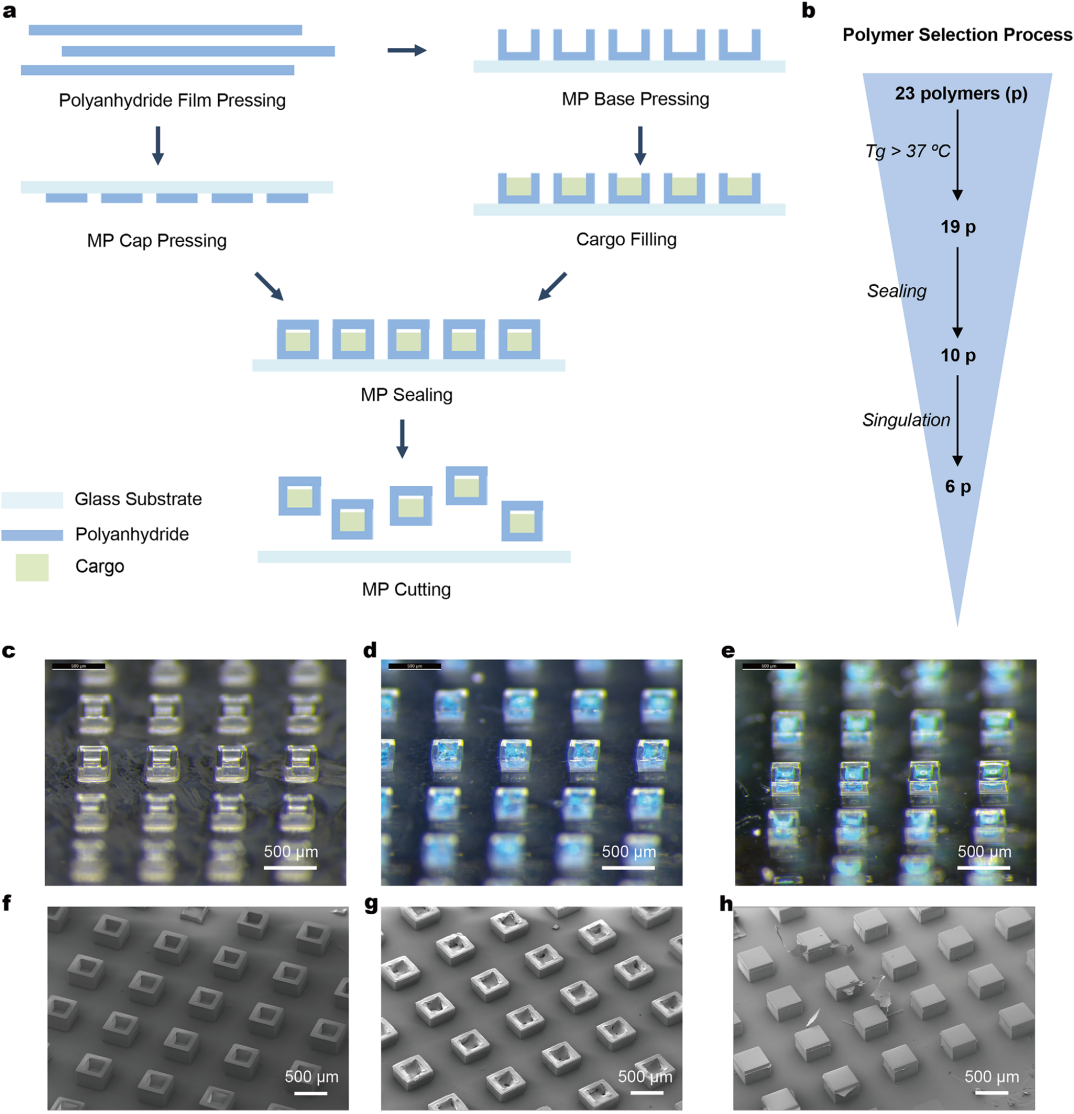
Material screening, fabrication performance, and microscopic characterization of polyanhydrides for SEAL core‐shell MPs. a) The SEAL core‐shell MPs were fabricated through a multi‐step process involving polymer film pressing, base and cap pressing, cargo filling, MP sealing, and MP singulation. b) An inverted funnel approach was employed to screen 23 initial polyanhydride compositions, narrowing down to 6 compositions that demonstrated optimal fabrication performance. c,d,e) Representative high‐resolution light microscopy images showing an array of empty bases pressed from polyanhydride polymer film (c), bases filled with DT antigen, excipients, and a dye (d), and sealed MPs (e). f,g,h) Representative scanning electron microscopy (SEM) images of empty bases (f), filled bases (g), and sealed MPs (h).

We synthesized and tested 23 representative polyanhydride compositions for SEAL‐based core‐shell MP fabrication, using an inverted funnel approach to narrow down the ideal candidates that are compatible with SEAL (**Figure** **2**b; Table S1, Supporting Information). The fabrication process involved hot‐pressing polymers into thin films, molding them into bases and caps, filling with cargo, sealing by heating above the polymer Tg, and singulating the microparticles for downstream use (Figure 2a).^[^ ^22^ ^]^ All of the selected polyanhydride compositions successfully formed polymer films and subsequently were compatible with the MP fabrication process, resulting in bases and caps with well‐defined and integral particle features (Figure 2c,f; Figure S3; Table S1, Supporting Information). Since the MPs will be used at body temperature for extended periods, the polyanhydride materials were required to have a Tg higher than 37 °C.^[^ ^44^ ^]^ Of the 23 selected compositions, 19 met this requirement (Figure 2b; Table S1, Supporting Information). The range of Tg for these materials was suitable for two reasons: first, the Tg values of the tested polyanhydride polymers were close to those of PLGA materials previously used for SEAL MP fabrication, falling within a range of 45 °C ± 10 °C; and second, these temperatures were tolerable for the cargo of interest, as the polymer needs to be heated to its Tg to complete the sealing step.^[^ ^22^ ^]^

All polymer compositions met the criteria for the cargo filling step, as this process depends on the physical geometry of the MPs rather than the specific material chemistry (Figure 2d,g; Figure S4, Supporting Information). The sealing step, however, presented challenges for some of the new materials, due to differences in alkyl chain length, which affected molecular packing and stability, and reduced the number of ideal composition candidates to 10 novel compositions (Figure 2b,e,h; Figure S5; Table S1, Supporting Information). Successful sealing was defined as the robust attachment of the bases to the caps, resulting in multilayered microstructures with a core that encapsulates the cargo. This process involved two sequential steps. First, the caps are heated just above the polymer's Tg, allowing them to soften and flow when brought into contact with the polymer bases. The heat flow must be sufficient to sinter the caps and bases effectively while minimizing contact time to avoid destabilizing the cargo. Second, the PDMS mold containing the caps – now attached to the bases – was removed and cooled to room temperature. The bases must remain securely attached to the caps, as delamination from the bases rather than the PDMS mold results in incomplete MPs. The sealed MPs were characterized using high‐resolution optical microscopy and scanning electron microscopy (SEM). Of the polyanhydrides tested, 10 compositions consistently achieved successful sealing, producing high‐fidelity, well‐aligned, cubic‐shaped MPs (Figure 2b, e, and h; Figure S5; Table S1, Supporting Information). Conversely, unsuccessful sealing led to various MP structures, including non‐sealed, half‐sealed, and pancake‐sealed microstructures. In cases of no sealing, no bases adhered to the caps even when significant pressure was applied to bring them into contact (Figure S6, Supporting Information). Ineffective heat flow caused the bases to become flattened or deformed upon contact with the caps. In other cases, the heat flow was sufficient, but sealing occurred in only some MPs or only when strong forces were applied, resulting in flattened or deformed bases and unsuccessful sealing (Figure S6, Supporting Information).

Interestingly, we observed a correlation between the length of the alkyl chain in the monomers and the success of the sealing process. Specifically, only the compositions with odd‐numbered alkyl chain lengths (n = 3, CPPr; n = 5, CPPe) consistently achieved successful sealing. Compositions with even‐numbered alkyl chain lengths (n = 6, CPH; n = 8, CPO) showed lower sealing success rates. This correlation was further supported by a progressive decrease in the sealing rate, transitioning from full sealing to partial sealing – where ≈50% of MPs were successfully sealed and the remainder remained open – and eventually to no sealing as the monomer feed ratio of p(o‐CPPr:p‐CPH) was adjusted from 100:0 to 50:50, and finally to 0:100 (Figure S7, Supporting Information). We hypothesize that the observed correlation between alkyl chain length and sealing success may be due to differences in molecular packing and interchain interactions associated with chain parity. To further test and validate this hypothesis, molecular dynamics (MD) simulations were performed on representative para substituted monomers with varying alkyl chain lengths: two odd numbered (n = 3, p CPPr; n = 5, p CPPe) and two even numbered (n = 6, p CPH; n = 8, p CPO). The degree of molecular aggregation was estimated using solvent accessible surface area (SASA) analysis, with lower SASA values indicating higher aggregation.^[^ ^53^ ^]^ At thermodynamic equilibrium, p CPPr and p CPPe exhibited significantly lower SASA values compared to p CPH and p CPO, suggesting stronger aggregation and tighter molecular packing for the odd numbered monomers (Figure S8, Supporting Information). Visual comparisons further confirmed that odd numbered monomers aggregate in a more compact and cohesive manner than their even numbered counterparts. Others have shown that odd‐numbered alkyl chains promote tighter molecular packing, creating a more cohesive and stable structure potentially conducive to effective sealing, while even‐numbered chains may result in less efficient packing due to alternating orientations in the polymer matrix.^[^ ^54^ ^]^

Following the screening process, we selected 6 compositions combining CPPr and CPPe monomers, with variations in the position of ring substituents and monomer ratios (Figure S9; Table S2, Supporting Information). These 6 compositions exhibited excellent fabrication performance, particularly with high sealing and singulation success rates exceeding 75%.

### 2.3 In Vitro Pulsatile Release of DT Vaccine via Polyanhydride‐Based Core‐Shell MPs

We selected DT as a model vaccine to evaluate the effectiveness of the polyanhydride‐based core‐shell MP platform due to its sensitivity to acidic environments. By using DT, this study aims to demonstrate the platform's capability to protect acid‐sensitive antigens and deliver them effectively in a single‐injection, self‐boosting vaccine format.

To assess the in vitro release kinetics of the single‐injection DT vaccine, we encapsulated the vaccine within core‐shell SEAL MPs fabricated using six polyanhydride compositions. Three commonly used vaccine excipients – trehalose, bovine serum albumin (BSA), and histidine – were evaluated for their ability to stabilize the DT vaccine within the MPs.^[^ ^41^ ^]^ The flexibility of loading various cargo compositions into an array of MPs enabled high‐throughput screening to identify an effective combination of excipients, underscoring the formulation advantage of the SEAL platform over traditional emulsion‐based MPs. We used one of the simplest and most studied polyanhydride compositions, p(o‐CPPr), for excipient formulation development. The three selected excipients – trehalose, BSA, and histidine – were tested individually and collectively within the MPs alongside the DT vaccine. Release of the active DT antigen was monitored for each MP at 37 °C, and the release of active antigen was measured by ELISA at various time points until reaching a plateau. Notably, when trehalose was used as the sole excipient, no active DT antigen was successfully stabilized or released. In contrast, formulations with BSA and histidine achieved 2.2% and 21.8% release of active DT antigen, respectively (Figure S10, Supporting Information). Interestingly, the combined formulation of all three excipients significantly enhanced stabilization, resulting in 89.7% release of active DT antigen (Figure S10, Supporting Information). In both the histidine‐only group and the group with all three excipients, release began one week after incubation. However, while the histidine‐only group showed no detectable active antigen after three days, the combined excipient formulation continued releasing active DT antigen for up to two weeks. These results suggest that excipient formulation plays a crucial role in determining the release time point.

With an optimized excipient formulation – comprising trehalose, BSA, and histidine – we fabricated core‐shell MPs with the DT vaccine using the six polyanhydride compositions selected from the screening studies (Figure S9, Supporting Information). A quality control step was first implemented to identify and exclude MPs that released antigen prematurely. Specifically, individual MPs were incubated at 37 °C in release buffer and assayed for DT release at Day 3. Any MP exhibiting measurable antigen release at this time point was classified as “early release” (i.e., likely due to incomplete sealing) and excluded from subsequent in vitro release analyses (Figure S11, Supporting Information). Then, the release kinetics profiles of the DT vaccine from individual MPs were monitored until reaching a plateau. To enable direct comparison between compositions, we constructed release kinetics profiles based on three key parameters for single‐injection vaccine applications: the 50% release time point, the 90% release time point, and the time window between these points, which corresponds to the level of pulsatile release. The 50% and 90% release points represent the middle and end points of DT antigen release, while the time window reflects the degree of pulsatile behavior.

**Figure 3:**
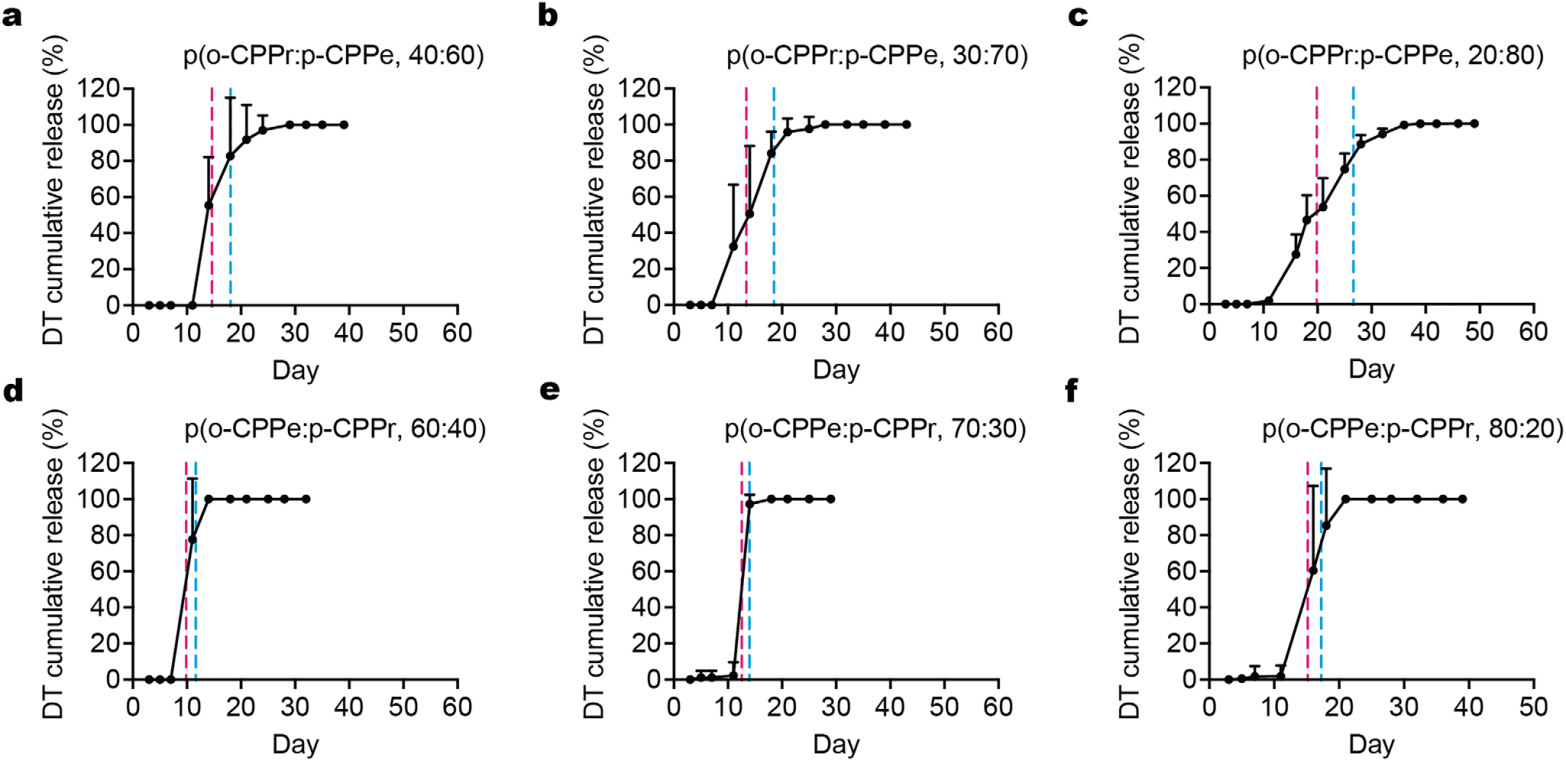
DT antigen in vitro release profiles of polyanhydride core‐shell MPs. Cumulative release of DT antigen as determined by ELISA in an in vitro environment for a) p(o‐CPPr:p‐CPPe, 40:60), b) p(o‐CPPr:p‐CPPe, 30:70), c) p(o‐CPPr:p‐CPPe, 20:80), d) p(o‐CPPe:p‐CPPr, 60:40), e) p(o‐CPPe:p‐CPPr, 70:30), and f) p(o‐CPPe:p‐CPPr, 80:20).

The release kinetics profiles were plotted based on cumulative release (**Figure** **3**; Table S3, Supporting Information). The middle release time points ranged from 9.8 days to 19.9 days, while the end points spanned from 11.6 days to 26.6 days. The earliest middle and end points were observed with p(o‐CPPe:p‐CPPr, 60:40), while the latest were seen with p(o‐CPPr:p‐CPPe, 20:80). Interestingly, the former composition exhibited the second shortest release window (1.8 days), while the latter exhibited the longest (6.7 days). The composition p(o‐CPPe:p‐CPPr, 70:30) delivered the narrowest release window at just 1.4 days. These results suggest a possible inverse correlation between the timing of release onset and the degree of pulsatile release behavior. The structure‐function relationship influenced by monomer composition was further explored using a machine learning model, as discussed in the following section. This platform shows potential for small, frequent vaccine dosages, which have been shown to provide greater potency than single bolus injections.^[^ ^55^ ^]^ Collectively, while the release profiles did vary across the six polyanhydride compositions, demonstrating that monomer identity and ratio can modulate both the onset and duration of antigen release, the current formulations produced a release onset within a relatively narrow window (10‐20 days). This suggests that achieving significantly longer boost intervals, such as over 1 month, may require additional strategies. Future modifications may include incorporating more hydrophobic polymer chemistries (e.g., using monomers with longer alkyl chains), crosslinking the polyanhydride chains to form a denser polymer network, or increasing the wall thickness of the MPs from the structural design perspective.

The final parameter evaluated for the polyanhydride core‐shell MPs was the cumulative recovery of active DT antigen, with recovery ranging from 39.7% to 89.7% relative to the initial loading upon fabrication, demonstrating successful DT vaccine release from polyanhydride‐based SEAL MPs (Table S3, Supporting Information). To further demonstrate the advantage of this platform over the PLGA‐based version in terms of pH stabilization, we first confirmed that the DT vaccine is indeed sensitive to acidic conditions. Its activity significantly decreased as the pH of the aqueous solution decreased (Figure S12, Supporting Information). PLGA degradation produces lactic and glycolic acids, which can lower the microenvironmental pH to as low as 1.5 within microspheres.^[^ ^56^ ^]^ In contrast, the pH of saturated aqueous solutions of typical polyanhydride degradation byproducts was measured to be 4.13 for o‐CPPr diacid and 6.30 for p‐CPH diacid, 2.8‐fold and 4.2 fold higher than that of PLGA, respectively. This internal acidification can be detrimental to the stability and compatibility of encapsulated molecules.^[^ ^27^ ^]^ To verify if this also occurs in the MPs, we encapsulated DT in a PLGA‐based SEAL MPs.^[^ ^22^ ^]^ We quantified the DT antigen recovery from PLGA‐based SEAL MPs at the expected release day and compared it with that of the six selected polyanhydride‐based MPs. Notably, the PLGA‐based MPs achieved only a 4.9% recovery, whereas the polyanhydride‐based MPs demonstrated an 8‐ to 18‐fold higher recovery rate, ranging from 39.7% to 89.7% (Figure S13, Supporting Information). Collectively, these findings indicate that polyanhydride‐based SEAL MPs successfully provided the necessary stabilization for the DT vaccine and yielded effective release due to its ability to maintain a less acidic pH environment in the core compared to the PLGA‐based SEAL MPs tested.

### 2.4 Machine Learning‐Based Modeling for Polyanhydride Core‐Shell MPs

While our results demonstrated the flexibility and tunability of the polyanhydride core‐shell MPs to achieve self‐boosting vaccination with desired release kinetics, significant bottlenecks remain in extending the general applicability of this platform. In theory, an infinite number of combinations could be generated by considering five key parameters related to material selection and the fabrication process: monomer type, feed ratio, molecular weight (MW) of the polyanhydride, vaccine loading amount, and excipients in the MPs. Screening such an extensive range of combinations would be labor‐intensive and time‐consuming, particularly when multiple factors must be evaluated simultaneously. Therefore, using a limited set of data points from in vitro release studies, we explored the potential of a machine learning (ML) model to predict correlations between these parameters and the performance of the MPs, as measured by release kinetics and antigen recovery.

**Figure 4:**
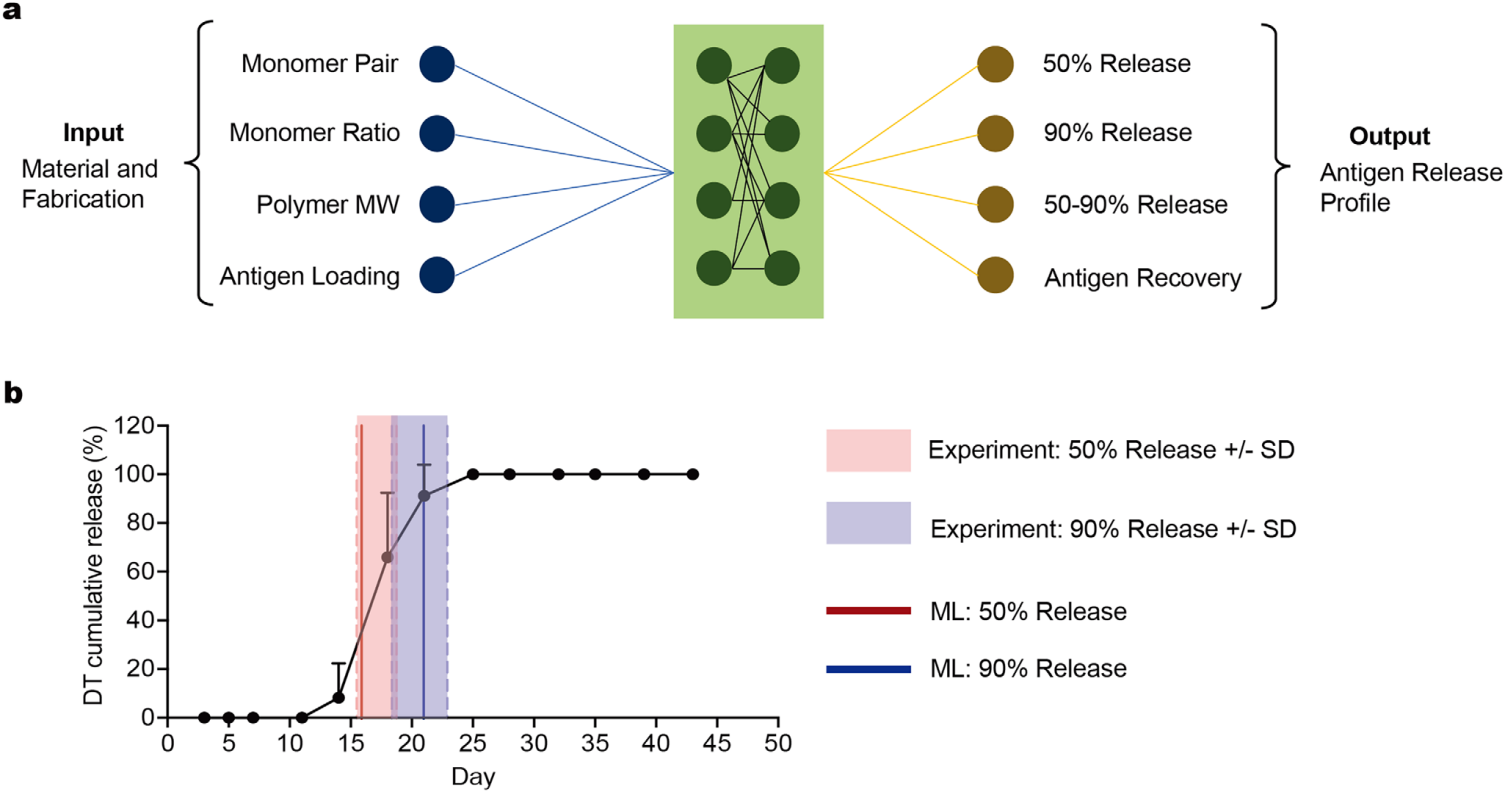
Machine learning modeling of polyanhydride core‐shell MP profiles. a) Four features related to material selection and fabrication were used as input to construct a machine learning model, with four antigen release profile features used as output. b) Machine learning‐predicted results for the 50% and 90% release time points were compared with experimentally obtained values, with predictions falling within one standard deviation of the experimental results.

The model employed four input parameters: monomer pair, monomer feed ratio, polymer MW, and vaccine loading amount (**Figure** **4**a; Figure S14; Tables S3,S4, Supporting Information). Four output features were evaluated: 50% release time point, 90% release time point, release time window, and antigen recovery (Figure 4a; Figure S14; Tables S3,S4, Supporting Information). Given that only six formulations were experimentally tested, we used the leave‐one‐out (LOO) cross‐validation method to develop the ML model.^[^ ^57^ ^]^ Additionally, we employed the least absolute shrinkage and selection operator (LASSO) method for variable selection (Figures S15 and S16, Supporting Information).^[^ ^58^ ^]^ The importance of each parameter was reflected by the absolute value of its weight coefficient, with positive values indicating a positive correlation and negative values indicating a negative correlation. The regression analysis revealed that the monomer structure and monomer ratio were the two most significant factors contributing to three output features: 50% release time point, 90% release time point, and release window (Figures S15 and S16, Supporting Information), which was expected given that these three parameters are interconnected. Interestingly, for total DT recovery, vaccine loading was identified as the most significant factor, although other factors also showed varying degrees of correlation. Based on these findings, we optimized the ML model to use only monomer structure and monomer ratio to predict the 50% release time point, 90% release time point, and release window, while retaining all four input variables for predicting total DT recovery.

Using this optimized ML model, we predicted release time points and antigen recovery for a wide range of combinations. We generated predictions for a total of 480 combinations based on a representative set of input features (Table S5, Supporting Information). This dataset enabled a mechanistic study of the correlations between fabrication parameters and release profiles. The modeling results indicated that, compared to the monomer pair o‐CPPr:p‐CPPe, o‐CPPe:p‐CPPr combinations yielded earlier release time points with smaller windows and lower antigen recovery (Figure S17, Supporting Information). As expected, increasing the ratio of the monomer with a longer alkyl chain delayed the release time point, while also extending the time window and increasing antigen recovery (Figure S18, Supporting Information). The Mw of the polymer was not predicted to significantly affect antigen recovery, while higher antigen loading in MPs led to decreased recovery at the release time point (Figures S19 and S20, Supporting Information). Consistent with these observations, the ML analysis indicated only a minor influence of MW on both release timing and antigen recovery (Figures S15 and S19, Supporting Information). This phenomenon is hypothesized to arise from the broad PDIs (≈2) of the polyanhydride polymers masking any MW‐dependent effects (Table S2, Supporting Information).

To validate the accuracy of the ML model in predicting release kinetics for combinations beyond the training set, we conducted an additional in vitro release study using MPs fabricated with the composition p(o‐CPPr:p‐CPPe, 30:70) and compared the experimental results with the LOO‐predicted values (Figure 4b; Tables S6 and S7, Supporting Information). The ML model predicted the 50% and 90% release time points for this composition to be at day 15.8 and day 21.0, respectively, with a release window of 4.8 days and 43.9% DT antigen recovery. Remarkably, the experimental results closely aligned with the ML‐predicted values. The predicted 50% and 90% release time points and recovery fell within one standard deviation of the experimental results, although the predicted release window was slightly larger. The model's predictive power could be further improved by expanding the number of data points.

In summary, we demonstrated the functionality of a preliminary ML‐based modeling approach, highlighting the potential of machine learning to design and optimize polyanhydride SEAL core‐shell MPs. In future applications, such modeling could be utilized to explore material selection, fabrication processes, and antigens in a rational and high‐throughput manner. Moreover, these models could help minimize batch‐to‐batch variability during MP fabrication, enhancing quality control. Future modeling efforts should also consider the feasibility of MP fabrication, which was manually screened in this study, but has the potential to be automated in future applications.

### 2.5 Self‐Boosting Vaccine by Programmed Pulsatile Release In Vivo

We next evaluated whether the six selected in vitro release profiles could be translated into an in vivo self‐boosting vaccine. Specifically, we sought to determine if the SEAL MP delivery platform could replace the multi‐dose requirement with a single injection in mouse models while generating an immune response comparable to the traditional multi‐dose regimen. Subcutaneous injection was chosen due to its widespread use in vaccine studies and its compatibility with the MP system.^[^ ^11^, ^44^ ^]^ Following subcutaneous administration, the core‐shell MPs remained at the injection site beneath the skin for weeks to months, depending on their degradation rate.

**Figure 5:**
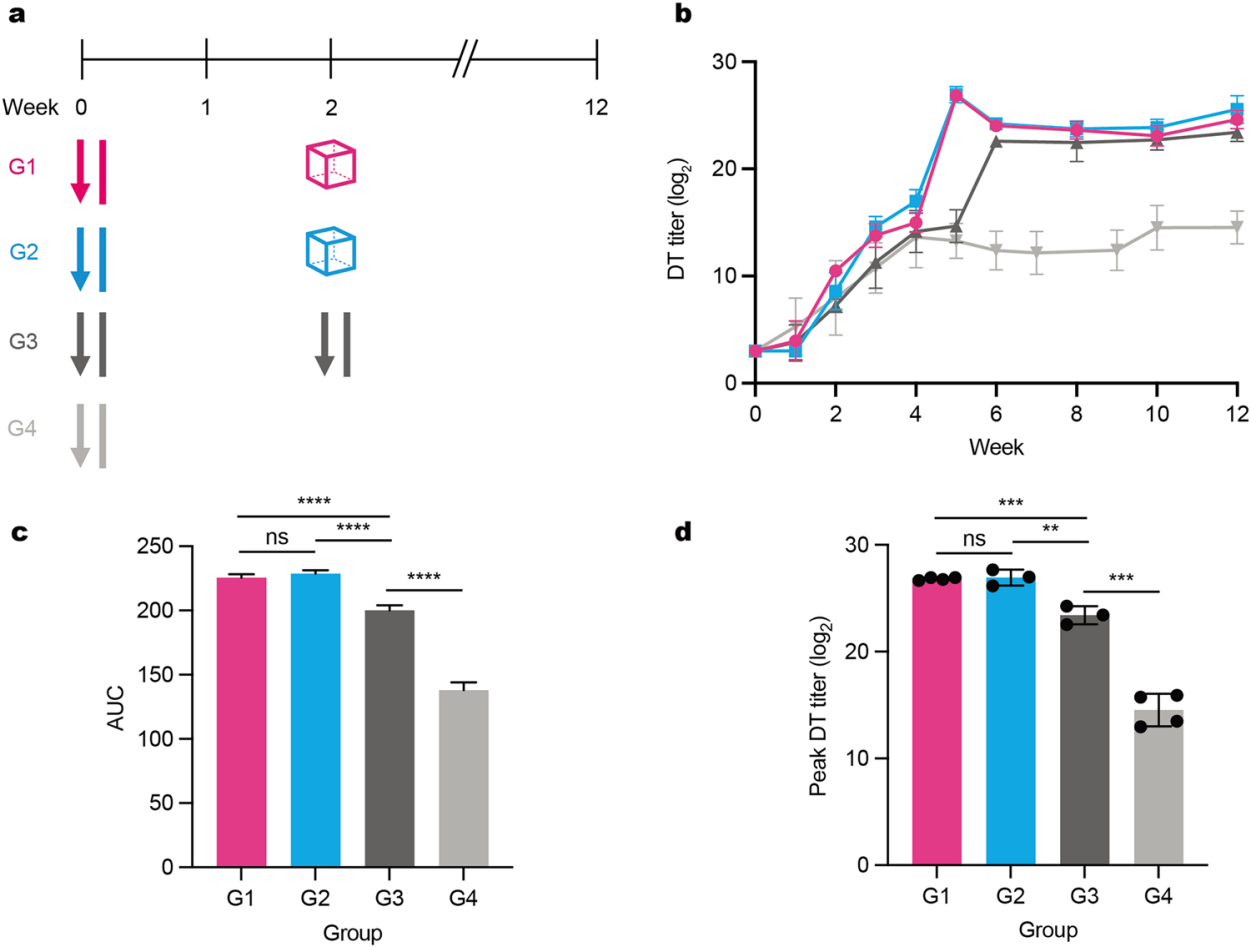
DT titer response of the polyanhydride core‐shell MP platform. a) Four groups were evaluated to compare in vivo delivery of DT antigen via conventional bolus injection and polyanhydride MP‐based single injection in the subcutaneous region. All groups received a prime bolus dose at the start of the study, with the booster dose designed for release at the 2‐week time point. b) ELISA results for DT‐specific titers showed that the two selected polyanhydride core‐shell SEAL MPs generated immune responses non‐inferior to the two‐bolus injection group and significantly higher than the single‐bolus injection group. c) Area under the curve (AUC) analysis of DT titer curves indicated that both polyanhydride MP groups achieved superior titer responses compared to the soluble injection control groups. d) Peak DT titer results also demonstrated that both polyanhydride MP groups outperformed the soluble groups. The data are presented as mean ± s.d. Statistical significance was evaluated using two‐tailed Student's *t*‐test. *P* ≤ 0.05 is statistically significant, with *P ≤ 0.05, **P ≤ 0.01, ***P ≤ 0.001 and ****P ≤ 0.0001.

To achieve a self‐boosting vaccine using the SEAL core‐shell MP platform, the antigen release time point should closely match the timing of a traditional booster injection, with a narrow release window being ideal.^[^ ^11^ ^]^ High antigen recovery at the release point is also crucial.^[^ ^11^ ^]^ In this study, the booster injection occurred two weeks after the prime dose. Among the six compositions tested in vitro, p(o‐CPPe:p‐CPPr, 70:30) and p(o‐CPPe:p‐CPPr, 80:20), were selected for in vivo testing. These compositions released DT antigen around the 2‐week time point, with narrow release windows (1.4 days and 2.1 days, respectively) and decent antigen recovery (39.7% and 50.7%, respectively). BALB/c mice were used in this study, and the animals were randomly assigned to four groups. Group 1 received a self‐boosting vaccine with p(o‐CPPe:p‐CPPr, 70:30), Group 2 received the p(o‐CPPe:p‐CPPr, 80:20) formulation, Group 3 received two bolus injections, and Group 4 received one bolus injection as a control (**Figure** **5**a). Specifically, in Week 0, Groups 1 and 2 received a prime dose of soluble DT vaccine along with a booster dose encapsulated in the corresponding SEAL MPs, designed to release their cargo two weeks post‐injection. Group 3 received a traditional multi‐dose vaccine regimen, consisting of a prime dose on Week 0 followed by a bolus booster dose on Week 2. Group 4, serving as the control, received only the prime bolus dose on Week 0, without any SEAL MPs or booster injection. Over the 12‐week study period, DT‐specific total IgG titers in mouse plasma were measured via ELISA to quantify the immune response.

Notably, the DT titer profiles in mice from Groups 1, 2, and 3 exhibited significant overlap, indicating that the SEAL MPs successfully achieved the programmed release at Week 2 (Figure 5b). In contrast, mice in Group 4, which did not receive a booster dose, showed no increase in DT titers after Week 4. The results showed that the DT titer increased ≈2–3 weeks after the booster dose, which was faster than the 4‑week rise observed following the primary injection. Interestingly, the DT titers in the SEAL MP groups (Groups 1 and 2) began to rise earlier than those in the soluble DT vaccine group (Group 3), possibly suggesting that some SEAL MPs released their cargo earlier than anticipated or that the presence of the MPs may have acted as an adjuvant, enhancing the response to the prime dose. The area‐under‐curve (AUC) analysis of DT titer profiles further confirmed that both polyanhydride MP groups (Groups 1 and 2) achieved immune responses superior to those of the soluble injection control groups (Figure 5c). Additionally, peak DT titer measurements demonstrated that the polyanhydride MP groups generated higher titers compared to the soluble injection groups (Figure 5d). Collectively, these results support the conclusion that the SEAL MP delivery platform can achieve pulsatile release at predetermined time points in vivo, with an immune response comparable to the traditional multi‐dose method and significantly superior to a single bolus injection.

## 3 Conclusion

In this study, we developed a polyanhydride‐based polymeric delivery platform capable of delivering a single‐injection self‐boosting vaccine, which holds potential for addressing global under‐immunization. From a broad library of possible formulations, six optimized polyanhydride core‐shell MPs were identified that successfully encapsulated DT vaccine and achieved pulsatile release at preset time points. The platform's effectiveness was demonstrated in both in vitro and in vivo settings, with immune responses comparable to traditional multi‐dose regimens. Additionally, we applied machine learning algorithms to optimize key parameters, including release kinetics and antigen recovery, enabling more efficient design and optimization for future applications. This work highlights the potential of the SEAL MP platform to simplify vaccine administration by reducing multi‐dose regimens to a single injection, offering a practical and scalable solution for enhancing immunization coverage worldwide.

## 4 Experimental Section

### Polyanhydride Synthesis

The polyanhydride polymers were synthesized via melt condensation polymerization, adopted from Conix et al. and Kipper et al.^[^ ^52^, ^59^ ^]^ Briefly, for the synthesis of p(o‐CPPr), to a 100 mL round bottom flask was added 2.0 g of o‐CPP monomer. While under vacuum, temperature was slowly increased from room temperature to 180 °C with constant stirring. After 90 min, the reaction was cooled down to room temperature followed by the addition of dichloromethane. The product was precipitated by gradual addition of hexane, collected by filtration, and dried under vacuum. Other polyanhydride compositions were synthesized following the same procedures with different feed monomers as starting materials.

### Fabrication of the Polyanhydride MPs

The Stamp Assembly of Polymer Layers process was used to fabricate the polyanhydride core‐shell MPs, as reported by McHugh, et al.^[^ ^22^ ^]^ Briefly, positive master molds of arrays of MP bases and caps were made by SU‐8 lithography on silicon wafers. Negative molds of the base and cap array features were created by PDMS base and curing agent. By pouring PDMS and curing agent mixture (9:1 w/w) between the positive master molds and a glass slide, the negative features were created after curing the mixture in an oven. Polyanhydride films were prepared via a heat pressing method, where the polymer powder was pressed in between two layers of Teflon films with a shim for thickness control. To mold the bases of MPs, a piece of polyanhydride film was pressed between the PDMS mold with base features and a clean glass slide and then heated for 2 h in a vacuum oven at 180 °C. This was then cooled to room temperature, and then the glass slide was peeled off from the PDMS mold, where the base array features were left on the glass substrate. The caps of MPs were fabricated in the same manner as the bases, with an extra layer of Teflon film inserted between the glass slide and the PDMS mold with cap features. After heating, the Teflon film and glass slide were removed away from the PDMS mold, where the polymer film stayed in the mold for later use. The cargo of interest was dissolved in water and then dispensed into the MP bases using cellenONE (Cellenion), a picoliter dispensing instrument. The amount of cargo to fill was controlled by concentration of the cargo aqueous phase and the number of cycles to dispense. To seal bases and caps, both arrays were first aligned under an optical microscope, and then the caps were briefly heated to the glass‐transition temperature of the polyanhydride, followed by sintering with the base array. The sealed MPs were then singulated from the glass substrate using a blade.

### In Vitro Release of DT Antigen from Polyanhydride Core‐Shell MPs

Temporal release of DT antigen from polyanhydride core‐shell MPs were characterized under in vitro environment. Briefly, one MP was placed in an Eppendorf tube filled with 1 mL of release buffer (PBS with 50 mM HEPES and 0.2% BSA). At each time point, 1 mL of the release buffer was removed and stored for quantification assay, followed by refilling of 1 mL of fresh release buffer. The samples were stored under 37 °C throughout the study. The amount of DT antigen in release buffer was quantified by enzyme‐linked immunosorbent assay (ELSA).

### Quantification of DT Antigen in Core‐Shell MPs

Enzyme‐linked immunosorbent assay (ELSA) was used to quantify the DT antigen in core‐shell MPs.^[^ ^60^, ^61^ ^]^ The ELISA was performed using 96‐well ELISA capture plates (Thermo Scientific). The plate was first coated with the monoclonal anti‐DT antibody in carbonate buffer by overnight incubation at 4 °C, followed by washing three times using PBS with 0.05% Tween 20. The plate was then blocked by incubation for 1 h at 37 °C with block buffer (PBS with 0.05% Tween 20 and 2.5% dry milk powder) followed by washing three times. DT antigen standards were prepared with 0.2 Lf mL^−1^ followed by serial dilution. The DT antigen samples were diluted to be within the range of standards. The DT antigen solutions were added to the plate and incubated for 1 h at 37 °C followed by washing three times. Next, the plate was incubated with the polyclonal guinea pig anti‐DT IgG solution for 1 h at 37 °C. After washing three times, the plate was then incubated with HRP goat anti‐guinea pig antibody for 1 h at 37 °C. The plate was washed again three times, then o‐Phenylenediamine dihydrochloride (OPD) in phosphate buffer with H_2_O_2_ was added and incubated at room temperature for 15 min. Sulfuric acid (6N) was then added, and the plate was read by a plate reader (Tecan) with wavelength of 492 nm.

### Serum Antibody Level Measurement after Immunization

All animal procedures were approved and performed under the guideline protocols (1019‐061‐22) of the Massachusetts Institute of Technology Committee on Animal Care. Female Balb/c mice aged 6–8 weeks were housed into four groups (n = 4). Two groups of the mice were injected with polyanhydride core‐shell MPs. Briefly, MPs containing a total of 0.2 Lf DT antigen were suspended in carboxymethyl cellulose (CMC) solution and then subcutaneously injected in the back of the mice, along with a prime dose of 0.2 Lf DT antigen. Two other groups served as controls. One control group, Group 3, was subcutaneously injected with 0.2 Lf DT antigen in PBS as prime dose, followed by a booster dosage injected in the same manner after 2 weeks. The other control group, Group 4, was only injected with 0.2 Lf DT antigen in PBS. Blood samples were collected from the mice weekly up to 6 weeks, then biweekly up to 12 weeks, following primary immunization injection. Test serum was collected by centrifugation and stored at −20 °C until analyzed by ELISA.^[^ ^61^ ^]^

### Quantification of Serum DT Antibody Level after DT Immunization

ELISA was used to measure antibody level of the serum samples.^[^ ^60^, ^61^ ^]^ The ELISA was performed in a similar manner as the ELISA protocol for DT antigen quantification from the in vitro studies. The plate was incubated overnight with DT antigen in carbonate buffer at 4 °C. The plate was then washed three times and then incubated with block buffer for 1 h at 37 °C. After an additional washing cycle, serum sample serially diluted in block buffer was added to the plate and incubated for 2 h at 37 °C. The plate was then washed six times and then anti‐mouse IgG HRP solution was added, followed by 1 h of incubation at 37 °C. The plate was subsequently washed six times again and then treated with OPD solution and sulfuric acid in the same manner as stated previously. The plate was then read by a plate reader (Tecan) at 492 nm.

### Machine‐Learning Modeling of MP Release Time and Antigen Recovery

The identity of the polymer was denoted by its design parameters, comprising monomer types, monomer ratios, the polymer's molecular weight, and vaccine loading, which served as the input features for the model. Monomer types (o‐CPPr:p‐CPPe and o‐CPPe:p‐CPPr) were represented as one‐hot encoded features. We developed models to predict the time of 50% release, 90% release, the window for 50%‐90% release, and the cumulative recovery. Prior to application, all features and prediction targets underwent standardization by removing the mean and scaling to unit variance.^[^ ^57^, ^58^ ^]^

We tested a variety of model architectures, including linear models, k‐nearest neighbors, support vector machines, Gaussian processes, random forests, gradient‐boosted decision trees, and neural networks. Model hyperparameters were tuned on a leave‐one‐out validation set, using the coefficient of determination (r^2) as the performance metric. Exhaustive feature combinations were tested, and it was determined that monomer type and ratio alone yielded the most accurate predictions for release time, whereas all features were necessary for accurate recovery predictions. The models that performed the best were linear models with l2 regularization (ridge regression) for predicting the 50% release and the 50%‐90% release window, a linear model with l1 regularization (Lasso) for predicting the 90% release, and a gradient‐boosted decision tree (XGBoost) for predicting recovery.

To discern the factors influencing release time and recovery, we conducted an analysis of the importance of each design parameter in our machine‐learning models. This investigation primarily relied on two types of models: lasso and random forest. In our lasso models, we leveraged the sparsity brought by L1 regularization and adjusted the regularization strength to identify the enduring features. For our random forest models, the impurity function of each node in the decision trees served as the metric for the corresponding feature's importance. In this context, the impurity function was defined as the mean squared error. The entire methodology was implemented using Python, with the models being realized in scikit‐learn and xgboost.

### Statistical Analysis

All quantitative characterizations were evaluated with at least three independent replicates. The data are presented as mean ± s.d. Statistical significance was evaluated using two‐tailed Student's *t*‐test. *P* ≤ 0.05 is statistically significant, with *P ≤ 0.05, **P ≤ 0.01, ***P ≤ 0.001, and ****P ≤ 0.0001.

## Conflict of Interest

For a list of entities with which R.L. is involved, compensated, or uncompensated, see: https://urldefense.com/v3/__https://www.dropbox.com/scl/fi/xjq5dbrj8pufx53035zdf/RL-COI-2024.pdf?rlkey=fwv336uoepiaiyg4e7jz5t4zo&dl=0__;!!N11eV2iwtfs!ve1zNWZL80Qgi-RaI7CpekrMlvn3YgZQJTReaSAZ0zVGw16iLBIFBGRV2vo8Ff_p5TrU-23QZXnAN0bXaEZp$. A.J. receives licensing fees (to patents on which she was an inventor) from, invested in, consults (or was on Scientific Advisory Boards or Boards of Directors) for, lectured (and received a fee), or conducts sponsored research at MIT for which she was not paid for the following entities: The Estée Lauder Companies; Moderna Therapeutics; OmniPulse Biosciences; Particles for Humanity; SiO_2_ Materials Science; VitaKey.

The remaining authors declare no competing interest.

## Supporting information



Supporting Information

## Data Availability

The data that support the findings of this study are available in the supplementary material of this article.
