# Supplementary material for: Polyanhydride‐Based Microparticles for Programmable Pulsatile Release of Diphtheria Toxoid (DT) for Single‐Injection Self‐Boosting Vaccines
Source: Adv Mater. 2025 May 15;37(32):2501168. doi: 10.1002/adma.202501168 (PMC12355444; doi:10.1002/adma.202501168)
Supplement: Supplementary file 1 — Supporting Information [file ADMA-37-2501168-s001.docx]

**Supplemental Information for**

**Polyanhydride-Based Microparticles for Programmable Pulsatile Release of diphtheria toxoid (DT) for Single-Injection Self-Boosting Vaccines**

Linzixuan Zhang^1^, Ruiqing Xiao^2^, Wenhao Gao^1^, Johnny Garcia^2^, Xinyan Pan^3^, John L. Daristotle^2^, Timothy Forster^2^, Jooli Han^2,6^, Mehr Chaddah^2^, Dhruv Varshney^2^, Nandita Menon^2^, Kevin J. McHugh^4, 5^, Benjamin J. Pedretti^1^, Jing Ying Yeo^1^, Xin Yang^2^, Sydney MacDonald^2^, Robert Langer^1,2†^, Ana Jaklenec^2†^

^1^Department of Chemical Engineering, Massachusetts Institute of Technology, Cambridge, MA 02139, USA.

^2^David H. Koch Institute for Integrative Cancer Research, Massachusetts Institute of Technology, Cambridge, MA 02139, USA.

^3^Department of Biological Engineering, Massachusetts Institute of Technology, Cambridge, MA 02139, USA.

^4^Department of Bioengineering, Rice University, Houston, TX 77005, USA.

^5^Department of Chemistry, Rice University, Houston, TX 77005, USA.

^6^Department of Biomedical Engineering, Seoul National University College of Medicine, Seoul, 03080, Republic of Korea

†Corresponding authors. Email: jaklenec@mit.edu (A.J.); rlanger@mit.edu (R.L.)

Keywords: Single-administration vaccines, protein encapsulation, microparticles, release kinetics, antigen stability

**Materials and Methods**

**Materials.** All materials were purchased from Sigma unless specified otherwise. The diphtheria toxoid, monoclonal anti-DT antibody, polyclonal guinea pig anti-DT IgG, HPR goat anti-guinea pig antibody were purchased from National Institute for Biological Standards and Control (NIBSC). The anti-mouse IgG HRP conjugate was purchased from Progema. PLGA was Evonik Resomer 502H.

**Synthesis of diacids.** The diacids were synthesized using methods adopted from works of Shen et al. and Conix et al.^1,2^ Briefly, for the synthesis of o-CPPr diacid, to a 500 mL round bottom flask was added salicylic acid (100 g, 0.72 mol, 2.0 eqv.) dissolved in 125 mL of H_2_O. While stirring, NaOH (72.5 g, 1.81 mol, 5.0 eqv.) was added slowly. The mixture was under reflux, followed by gradual addition of 1,3-dibromopropene (36.7 mL, 0.36 mol, 1.0 eqv.). The reaction was reflux overnight and subjected to distillation under reduced pressure. The solid was resuspended in 800 mL of H_2_O followed by addition of H_2_SO_4_ to pH of 3. The precipitate was isolated by filtration and dried under vacuum. The product was purified via recrystallization by dissolving the precipitate in ethyl acetate followed by gradual addition of hexane. Other diacids were synthesized following the same procedures using different hydroxybenzoic acids and dibromides as starting materials. The structures of products were characterized by nuclear magnetic resonance (NMR).

o-CPP: ^1^H NMR (500 MHz, DMSO-*d_6_*) δ 12.57 (s, 2H), 7.63 (dd, *J* = 7.6, 1.8 Hz, 2H), 7.48 (ddd, *J* = 8.8, 7.3, 1.8 Hz, 2H), 7.14 (d, *J* = 8.3 Hz, 2H), 6.99 (td, *J* = 7.5, 0.9 Hz, 2H), 4.25 (t, *J* = 6.2 Hz, 4H), 2.17 (t, *J* = 6.2 Hz, 2H).

p-CPPe: ^1^H NMR (500 MHz, DMSO-*d_6_*) δ 12.60 (s, 2H), 7.91 – 7.84 (m, 4H), 7.05 – 6.98 (m, 4H), 4.07 (t, *J* = 6.4 Hz, 4H), 1.81 (p, *J* = 6.7 Hz, 4H), 1.58 (qd, *J* = 8.8, 5.9 Hz, 2H).

**Synthesis of prepolymers/monomers.** The prepolymers/monomers were synthesized using methods adopted from works of Shen et al. and Conix et al.^1,2^ Briefly, for the synthesis of o-CPPr prepolymer, to a 500 mL round bottom flask was added o-CPPr diacid (10 g, 0.03 mol, 1 eqv.) followed by acetic anhydride (120 mL, 1.27 mol, 40 eqv.). The reaction was stirred under reflux for 30 minutes followed by distillation to remove all liquids. The product was further dried under vacuum. Other prepolymers were synthesized following the same procedures using different diacids as starting material. The structures of products were characterized by nuclear magnetic resonance (NMR).

o-CPPe: ^1^H NMR (500 MHz, CDCl_3_) δ 7.87 (dd, *J* = 7.8, 1.8 Hz, 2H), 7.55 (ddd, *J* = 8.9, 7.3, 1.8 Hz, 2H), 7.05 – 6.97 (m, 4H), 4.12 (t, *J* = 6.3 Hz, 4H), 2.32 (s, 6H), 2.00 – 1.89 (m, 2H), 1.82 – 1.72 (m, 2H).

p-CPPr: ^1^H NMR (500 MHz, CDCl_3_) δ 8.03 (d, *J* = 8.9 Hz, 12H), 6.99 (d, *J* = 8.8 Hz, 2H), 4.28 (t, *J* = 6.0 Hz, 4H), 2.39 (s, 6H), 2.36 (p, *J* = 6.0 Hz, 2H).

**Characterizations of polyanhydrides.** ^1^H NMR spectra were obtained for the monomers and polymers using Bruker Avance Neo spectrometer operating at 500.34 MHz for ^1^H(^13^C), in CDCl_3_ or DMSO-*d_6_*, and referenced to the residual solvent signal. Spectra for quantitative integration were recorded using 64 scans. The molecular weight (MW) of polymers were determined using an EcoSEC Elite gel permeation chromatograph (GPC) system with chloroform as the mobile phase. The elution times of the polymer samples were compared with polystyrene standards to determine MW. NMR spectra were obtained using Bruker Avance Neo spectrometer operating at 500.34 MHz for ^1^H(^13^C), in CDCl_3_, and referenced to the residual solvent signal. Thermal transitions were determined by differential scanning calorimetry (DSC) using a Discovery DSC from TA instruments with powdered samples (5−8 mg) sealed in aluminum pans.

P(oCPPr_CPPe20_80): ^1^H NMR (500 MHz, ) δ 8.07 (dd, *J* = 24.5, 8.6 Hz, 136H), 7.97 – 7.84 (m, 18H), 7.57 – 7.42 (m, 18H), 6.96 (dd, *J* = 27.3, 8.4 Hz, 176H), 4.09 (dt, *J* = 19.7, 6.0 Hz, 176H), 2.36 (s, 6H), 2.13 – 1.99 (m, 19H), 1.93 (p, *J* = 6.8 Hz, 144H), 1.71 (dh, *J* = 12.4, 6.0 Hz, 71H).

**Characterizations of the polyanhydride MPs.** Open base arrays, filled base arrays, and sealed core-shell MPs were characterized by scanning electron microscopy (SEM; Zeiss). The samples were coated with gold prior to the SEM imaging. The sample sets were also imaged by high-resolution optical microscopy (Leica).

**Cargo loading of the polyanhydride MPs.** Cargo filling was realized by cellenONE (Cellenion). Before the filling step, stock solutions were prepared for all components in the core-shell MPs, including DT antigen (4000 Lf/mL), trehalose (300 mg/mL), bovine serum albumin (BSA; 50 mg/mL), histidine (25 mg/mL), and an Alexa Fluor 647-labeled dextran dye (10 mg/mL). In each core-shell MP, the loading parameter was 0.03 Lf of the DT antigen, 4200 ng of trehalose, 2100 ng of BSA, 2100 ng of histidine, and 100 ng of the dextran dye.

**Molecular dynamics (MD) simulations.** MD simulations were carried out using the GROMACS software package.^3^ The force field parameters for the p-CPO, p-CPPr, p-CPH, and p-CPPe monomer molecules were assigned based on the General Amber Force Field (GAFF).^4,5^ Atomic partial charges were derived from quantum chemical calculations using ORCA software at the level of B3LYP/def2-SVP and fitted using the restrained electrostatic potential (RESP) method.^6–9^ Each system was constructed by placing 50 identical molecules in a cubic simulation box with dimensions of 5 × 5 × 5 nm³. The three dimensions were applied periodic boundary conditions (PBC). Long-range electrostatic interactions were computed using the particle mesh Ewald (PME) method, and all covalent bonds involving hydrogen atoms were constrained using the SHAKE algorithm.^10,11^ A cutoff distance of 10 Å was applied for non-covalent interactions.

Before the MD simulations, all systems were subjected to thorough energy minimization to eliminate unfavorable atomic configurations. Subsequently, 20 ns of simulations were performed in the canonical (NVT) ensemble to investigate the intermolecular interactions and self-assembly behavior of the molecules. All systems reached thermodynamic equilibrium within 2 ns, and trajectory analyses were conducted using the equilibrated portion of the simulations.

To further elucidate the intermolecular interactions and aggregation behavior of related molecules, additional simplified MD simulations were performed using systems composed of only four molecules. This setup allows for a more detailed analysis of specific interaction patterns, such as π–π stacking, and spatial arrangement, without the complexity introduced by large-scale aggregation.

**Supplemental Figure**


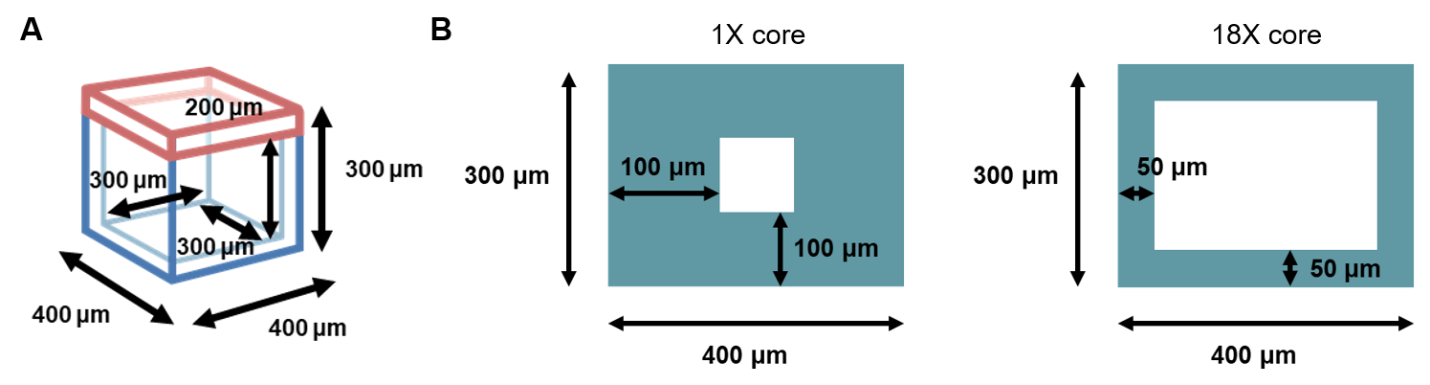


**Figure S1. Schematic and dimension of a SEAL core-shell microparticle (MP). (A)** Exterior and interior dimension of a SAEL core-shell microparticle. **(B)** Dimension comparison of the core-shell MPs between the original 1x and new 18x designs.

**Figure S2. Synthesis route of an exemplary composition, p(o-CPPr), in the aromatic polyanhydride library. (A)** Alkylation of salicylic acid and 1,3-dibromopropane to obtain the o-CPPr diacid. **(B)** Acetylation of the o-CPPr diacid with acetic anhydride to obtain the o-CPPr monomer. **(C)** Melt condensation polymerization of the o-CPPr monomer to obtain p(o-CPPr).


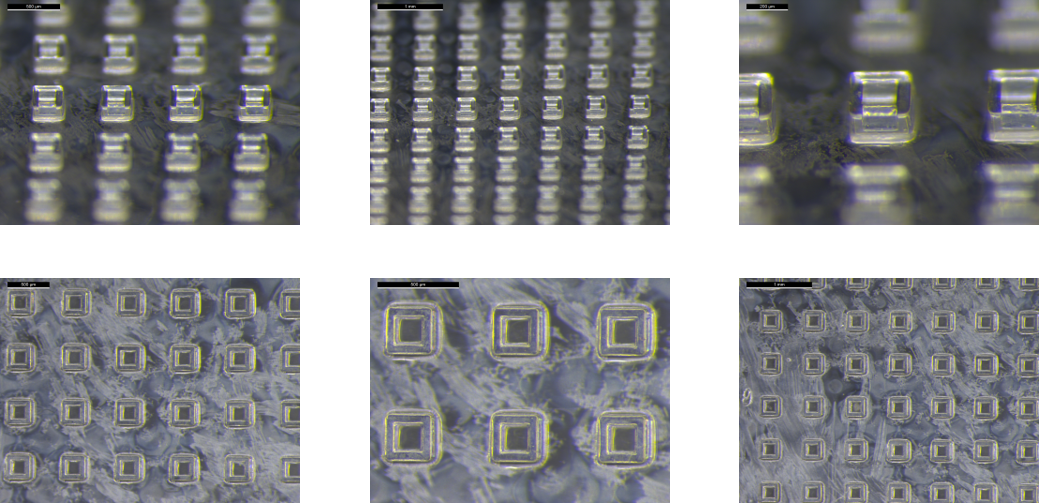


**Figure S3. Optical microscope images of MP base arrays.** Optical images of representative bases of the polyanhydride MPs were obtained using a high-resolution light microscope.


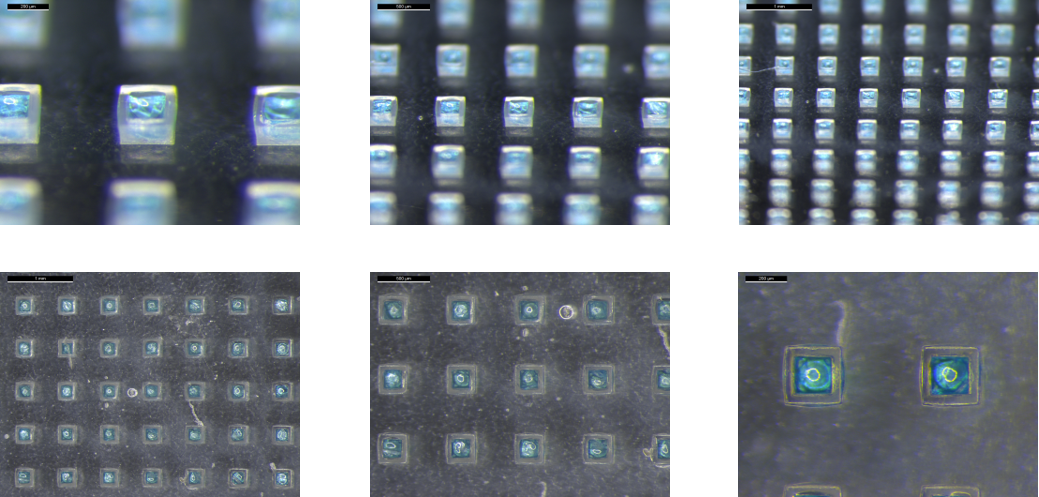


**Figure S4. Optical microscope images of MP cargo-filled base arrays.** A high-resolution light microscope was used to capture representative bases of the polyanhydride MPs filled with DT antigen, excipients, and a blue dye.


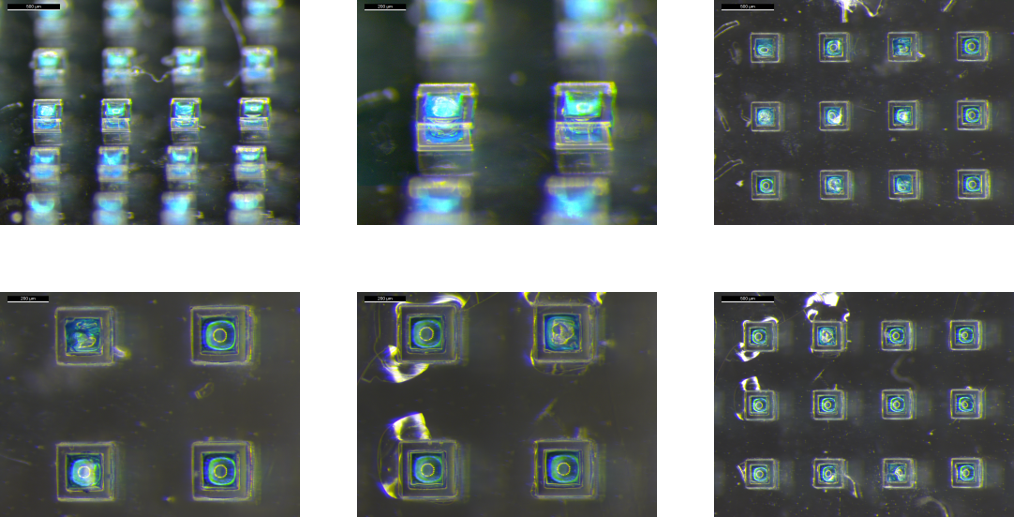


**Figure S5. Optical microscope images of sealed polyanhydride MPs.** The arrays of sealed polyanhydride MPs were imaged using a high-resolution light microscope.


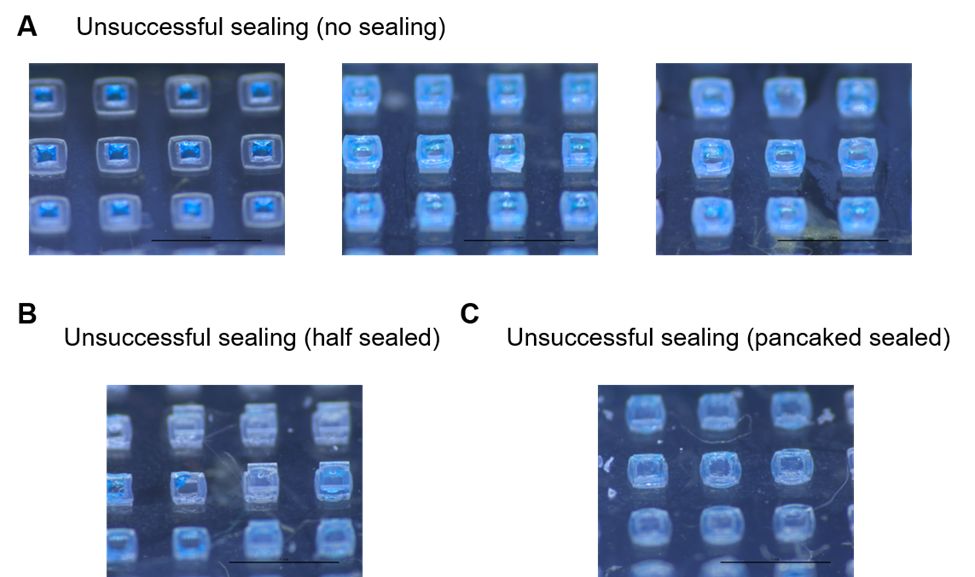


**Figure S6. Polyanhydride bases with unsuccessful cap sealing.** Representative optical images of polyanhydride base arrays after unsuccessful sealing attempts. **(A)** No caps were sintered to the bases. **(B)** Only some bases were successfully caped. **(C)** Bases were sealed with caps but were heated and pressed into pancake shape.


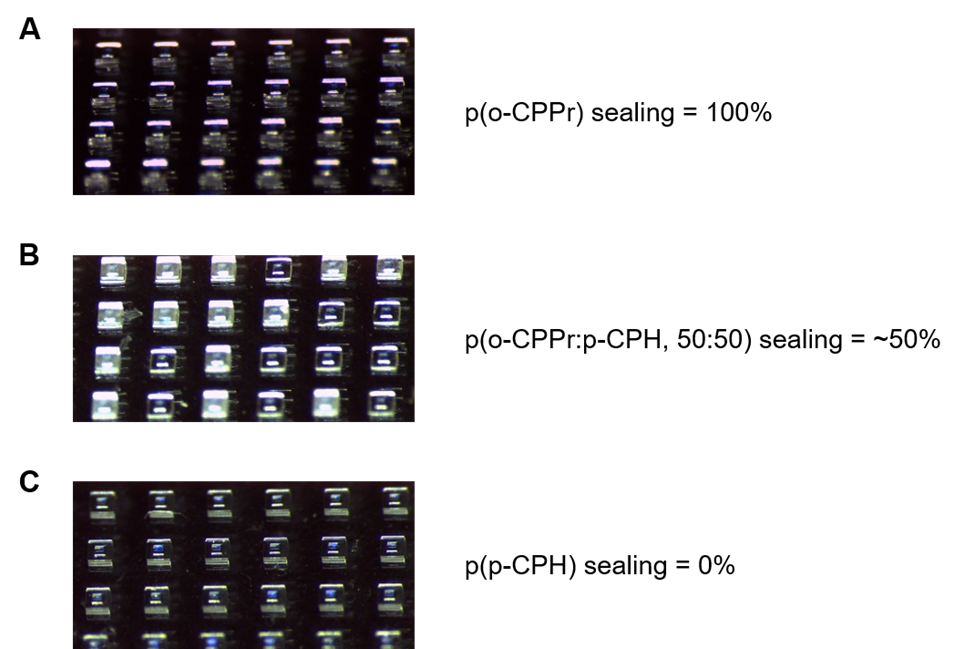


**Figure S7. Correlation between sealing rate and ratio of p-CPH in the polyanhydride polymer.** The successful rate of MP sealing decreased from 100% when p(o-CPPr) was used **(A)** to around 50% when p(o-CPPr:p-CPH, 50:50) was used **(B)**, and finally to no sealed MPs were obtained when p(o-CPH) was used **(C)**.


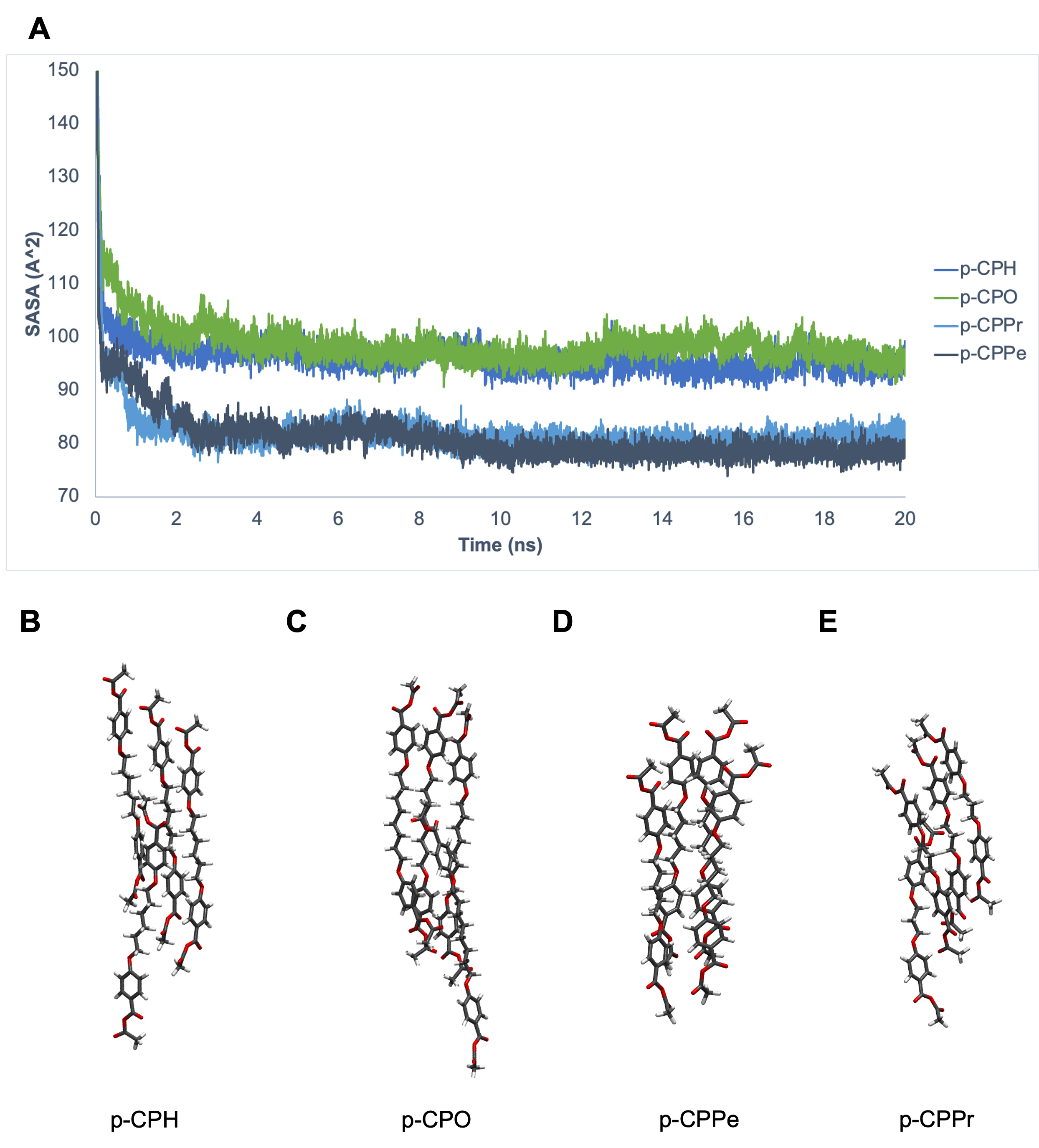


**Figure S8. Molecular dynamics simulation of polyanhydride monomers.** (A) Solvent-accessible surface area (SASA) analysis of four representative para-substituted monomers with varying alkyl chain lengths: two odd-numbered (n=3, p‑CPPr; n=5, p‑CPPe) and two even-numbered (n=6, p‑CPH; n=8, p‑CPO). Lower SASA values indicate stronger molecular aggregation. (B–E) Representative snapshots from MD simulations showing intermolecular interactions among four identical monomers in one simulation setting: p‑CPH (B), p‑CPO (C), p‑CPPe (D), and p‑CPPr (E).


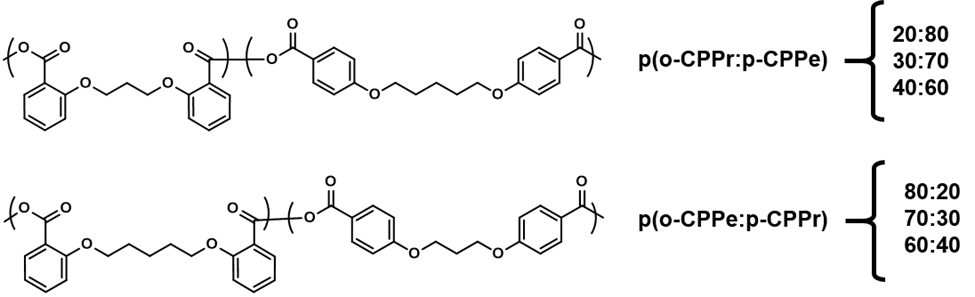


**Figure S9. Selected polyanhydrides for optimized MP fabrication process.** Structure of the six polyanhydride compositions were screened out for core-shell MP fabrication after optimization.


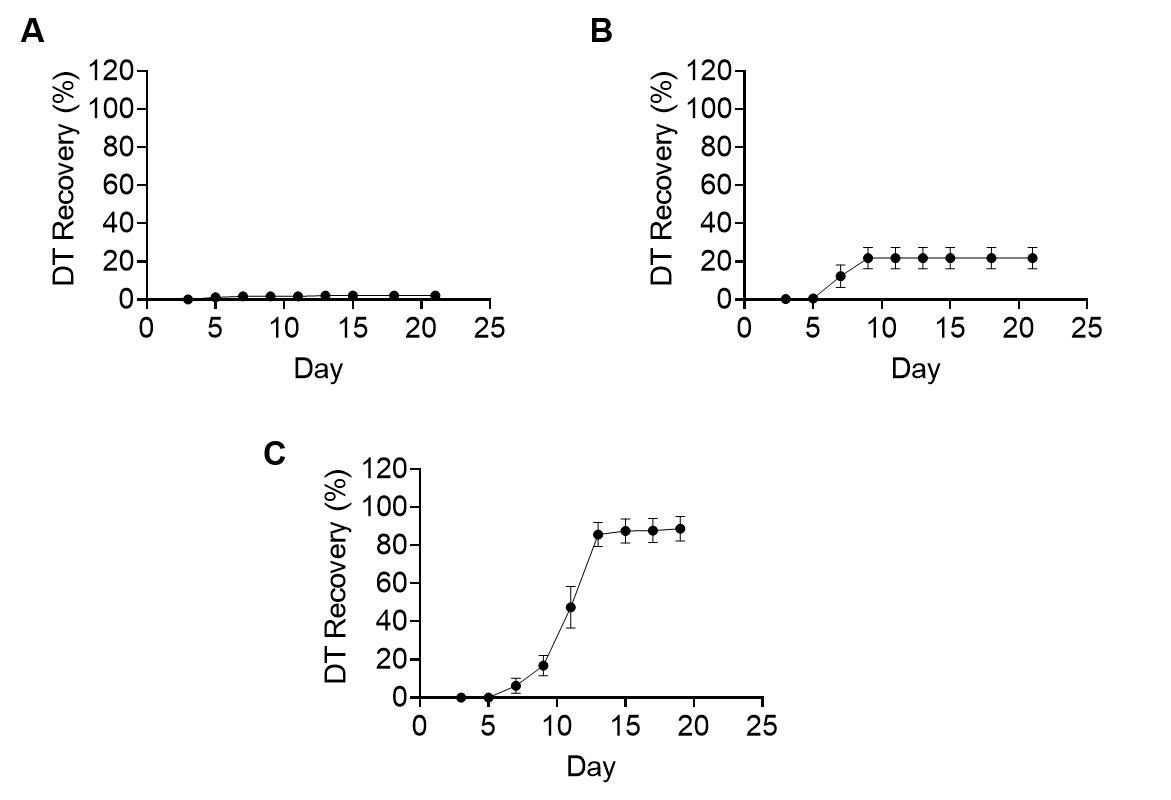


**Figure S10. DT recovery profiles from MPs with various excipient combinations.** The release profiles of recovered DT antigen from polyanhydride core-shell MPs under *in vitro* environment when **(A)** BSA only, **(B)** histidine only, and **(C)** a combination of trehalose, BSA, and histidine were used for antigen stabilization.

**Figure S11. Percentage of early‑release MPs.** Core‑shell MPs fabricated from the six selected polyanhydride compositions were incubated under the same conditions used for in vitro release studies and assessed at Day 3. MPs exhibiting burst release of DT antigen by Day 3 were classified as “early release,” and the proportion of these MPs was calculated as a percentage.

**Figure S12. The pH sensitivity of DT antigen in aqueous solution.** The DT antigen activity decreased as the acidity of aqueous solution increased.

**Figure S13. Comparison of DT antigen recovery in PLGA- and polyanhydride-based SEAL MPs.** The percentage of DT antigen recovery from SEAL MPs at the time of release was compared for PLGA and six selected polyanhydrides.


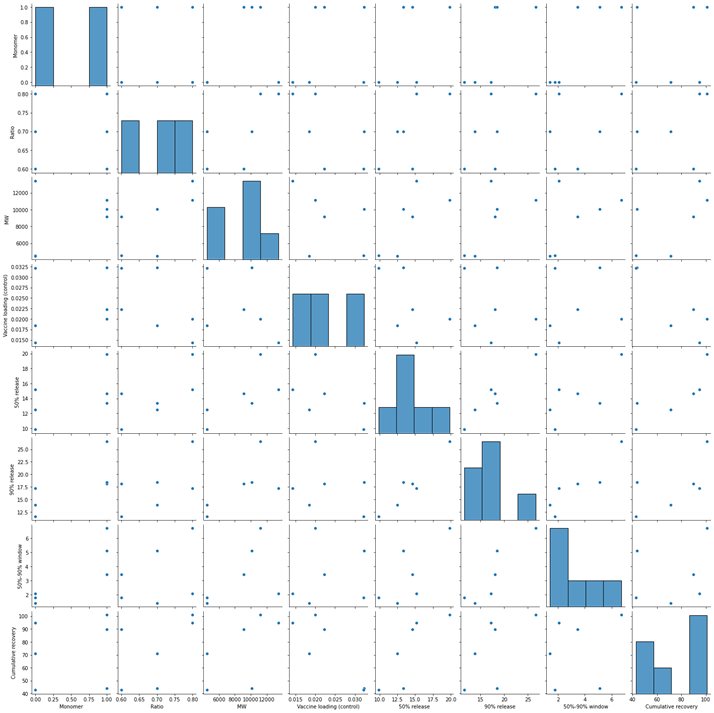


**Figure S14. Machine-learning (ML) dataset visualization.** The dataset composed of polyanhydride core-shell MPs release profiles under *in vitro* environment was plotted for visualization. These data points were used for machine-learning modeling.


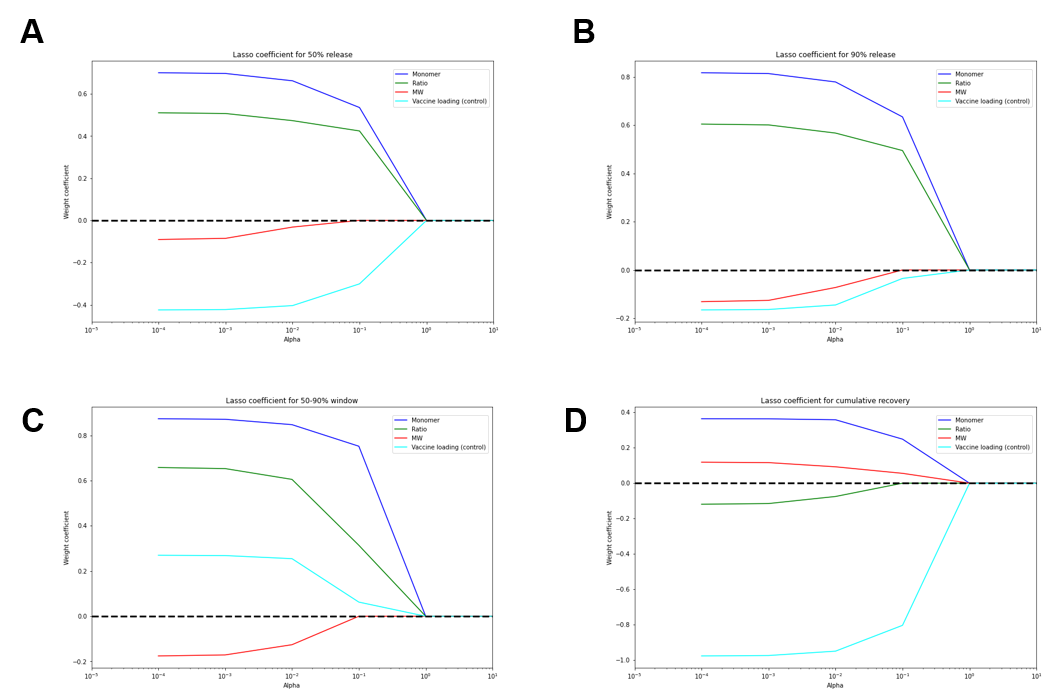


**Figure S15. LASSO coefficients of the ML model.** LASSO coefficients of the four input parameters, including monomer pair, monomer ratio, polymer MW, and vaccine loading were plotted for the four output parameters, which are **(A)** 50% release time point, **(B)** 90% release time point, **(C)** release time window, and **(D)** antigen cumulative recovery.


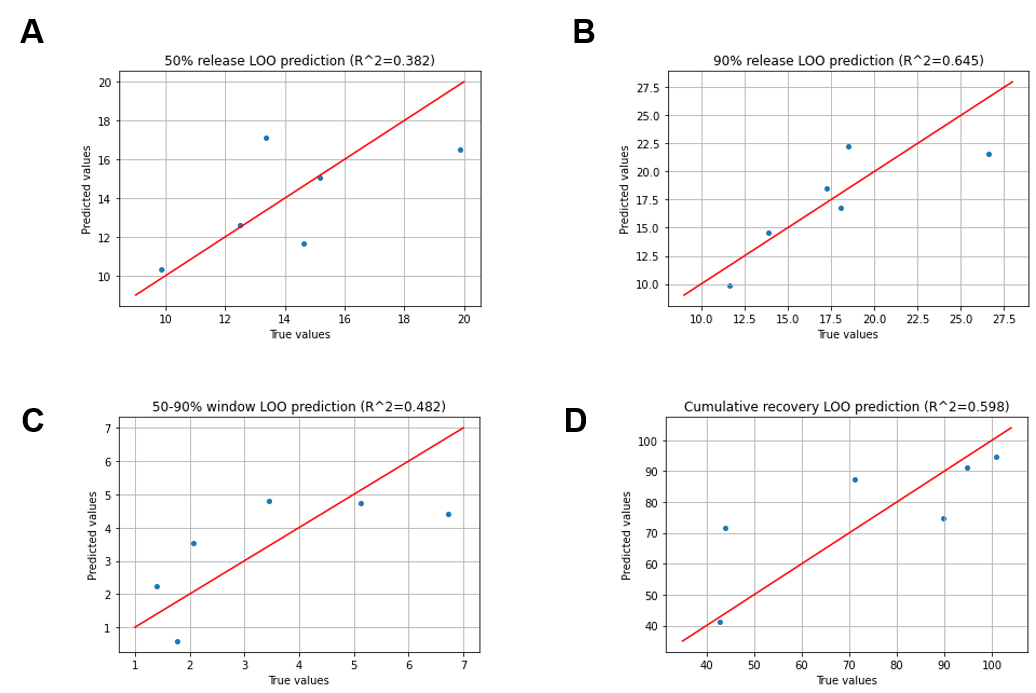


**Figure S16. LOO prediction and true values.** The linear correlation of LOO-predicted and true values was evaluated for the four output parameters, including **(A)** 50% release time point, **(B)** 90% release time point, **(C)** release time window, and **(D)** antigen cumulative recovery.


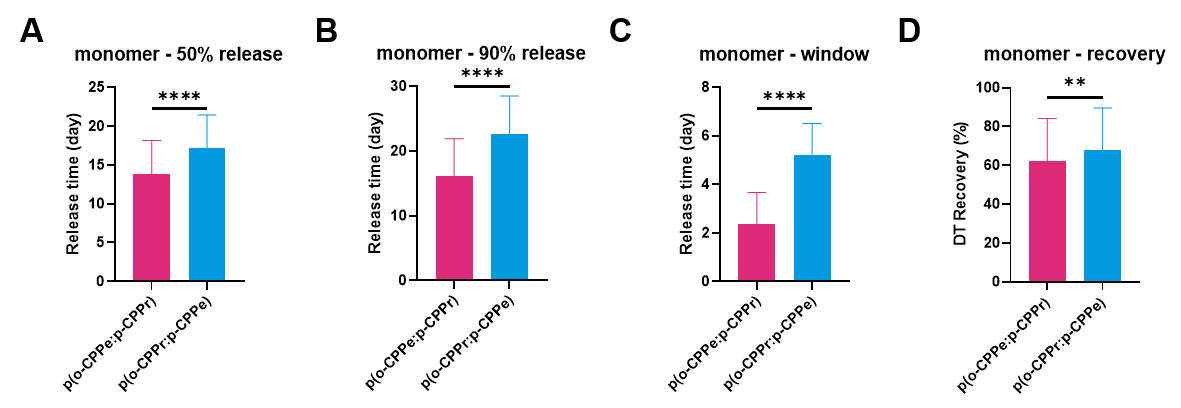


**Figure S17. Effect of monomer pair on antigen release and recovery based on ML prediction.** The 50% **(A)** and 90% **(B)** release time points were more delayed for the pair of o-CPPr:p-CPPe, which released the antigen in a larger time window **(C)**. The pair of o-CPPr:p-CPPe also showed higher DT antigen recovery in the ML model **(D)**.


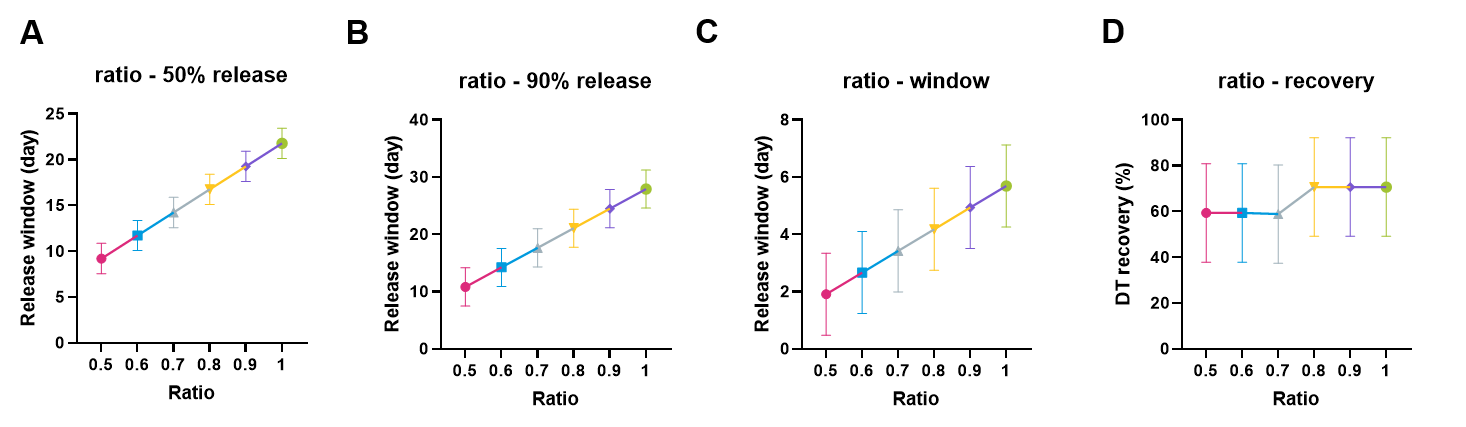


**Figure S18. Effect of monomer ratio on antigen release and recovery based on ML prediction.** The parameters of 50% release **(A)**, 90% release **(B)**, and the release time window **(C)** presented a positive correlation with the monomer ratio. Higher antigen recovery was predicted for then high ratio group (0.8 to 1) than the lower one (0.5 to 0.7) **(D)**.

**Figure S19. Effect of polymer MW on antigen release window based on ML prediction.** The low MW group (3000 to 5000) showed an increase correlation with MW, while passing the threshold of 8000 gave no correlation.

**Figure S20. Effect of vaccine loading on antigen recovery based on ML prediction.** Low antigen loading groups (0.01 to 0.02) showed high recovery, followed by a decrease trend with increasing antigen loading (0.03), and then plateaued at high loading groups (0.04 to 0.05).

**Supplemental Table**

**Table S1. Polyanhydride compositions tested for core-shell MP fabrication.**

| **Polymer** | **Monomer feed ratio** | **Tg (°C)** | **Sealing** |
| --- | --- | --- | --- |
| p(o-CPPr) | N/A | 52.7 | ✓ |
| p(o-CPPe) | N/A | 38.2 | ✓ |
| p(m-CPH) | N/A | 27.8 |  |
| P(o-CPPr:p-CPH) | 75:25 | 49.8 |  |
| P(o-CPPr:p-CPH) | 50:50 | 48.6 |  |
| P(o-CPPr:p-CPH) | 25:75 | 47.1 |  |
| p(o-CPPr:p-CPPe) | 50:50 | 51.0 | ✓ |
| p(o-CPPr:p-CPPe) | 40:60 | 59.0 | ✓ |
| p(o-CPPr:p-CPPe) | 30:70 | 58.2 | ✓ |
| p(o-CPPr:p-CPPe) | 20:80 | 56.9 | ✓ |
| p(o-CPPe:p-CPPr) | 50:50 | 51.3 | ✓ |
| p(o-CPPe:p-CPPr) | 60:40 | 58.0 | ✓ |
| p(o-CPPe:p-CPPr) | 70:30 | 45.1 | ✓ |
| p(o-CPPe:p-CPPr) | 80:20 | 60.0 | ✓ |
| P(o-CPPe:p-CPH) | 75:25 | 40.4 |  |
| P(o-CPPe:p-CPH) | 50:50 | 43.5 |  |
| P(o-CPPe:p-CPH) | 25:75 | 48.3 |  |
| P(o-CPPr:m-CPH) | 50:50 | 42.5 |  |
| p(p-CPH) | N/A | 47.0 |  |
| p(o-CPO) | N/A | 22.3 |  |
| P(o-CPO:p-CPH) | 75:25 | 28.4 |  |
| P(o-CPO:p-CPH) | 50:50 | 34.1 |  |
| P(o-CPO:p-CPH) | 25:75 | 40.7 |  |

**Table S2. Molecular weight information of the selected polyanhydride for core-shell MP fabrication.**

| **Monomer pair** | **Ratio** | **Mn** | **Mw** | **PDI** |
| --- | --- | --- | --- | --- |
| p(o-CPPr:p-CPPe) | 40:60 | 9131 | 19091 | 2.09 |
|  | 30:70 | 10084 | 21892 | 2.17 |
|  | 20:80 | 11167 | 24974 | 2.24 |
| p(o-CPPe:p-CPPr) | 60:40 | 4513 | 8521 | 1.89 |
|  | 70:30 | 4503 | 8899 | 1.98 |
|  | 80:20 | 13434 | 26772 | 1.99 |

**Table S3. Release kinetics information of the selected polyanhydride core-shell MPs.**

| **Monomer pair** | **Ratio** | **50% release** | **90% release** | **DT antigen recovery** |
| --- | --- | --- | --- | --- |
| p(o-CPPr:p-CPPe) | 40:60 | 14.7 days | 18.1 days | 89.7% |
|  | 30:70 | 13.4 days | 18.5 days | 43.8% |
|  | 20:80 | 19.9 days | 26.6 days | 69.4% |
| p(o-CPPe:p-CPPr) | 60:40 | 9.8 days | 11.6 days | 42.7% |
|  | 70:30 | 12.5 days | 13.9 days | 39.7% |
|  | 80:20 | 15.2 days | 17.3 days | 50.7% |

**Table S4. Polyanhydride SEAL core-shell MP datasets used for ML model building.**

| **Monomer** | **Ratio** | **MW** | **Vaccine loading** |
| --- | --- | --- | --- |
| o-CPPr:p-CPPe | 40:60 | 9131 | 0.0222 |
| o-CPPr:p-CPPe | 30:70 | 10084 | 0.0323 |
| o-CPPr:p-CPPe | 20:80 | 11167 | 0.0200 |
| o-CPPe:p-CPPr | 60:40 | 4513 | 0.0322 |
| o-CPPe:p-CPPr | 70:30 | 4503 | 0.0185 |
| o-CPPe:p-CPPr | 80:20 | 13434 | 0.0143 |

**Table S5. Input features to the ML model for prediction of antigen release and recovery from polyanhydride core-shell MPs.**

| **Parameter** | **Input feature** | |
| --- | --- | --- |
| **Monomer** | o-CPPe:p-CPPr | o-CPPr:p-CPPe |
| **Ratio** | 50:50; 60:40; 70:30; 80:20; 90:10; 100:0* | 50:50; 40:60; 30:70; 20:80; 10:90; 0:100 |
| **MW** | 3000; 5000; 8000; 10000; 13000; 15000; 18000; 20000 | |
| **Vaccine loading** | 0.01; 0.02; 0.03; 0.04; 0.05 | |

**Table S6. Input features of the ML model testing group of polyanhydride core-shell MPs.**

| **Input parameter** | **Input feature** |
| --- | --- |
| **Monomer** | o-CPPr:p-CPPe |
| **Ratio** | 70:30 |
| **MW** | 19654 |
| **Vaccine loading** | 0.032 |

**Table S7. Comparison between output results from the ML model and experimentally obtained results.**

| **Output parameter** | **Output results** | **Experimental results** |
| --- | --- | --- |
| **50% release** | 15.877 days | 17.112 +/- 1.618 days |
| **90% release** | 20.961 days | 20.618 +/- 2.281 days |
| **Window** | 4.847 days | 3.506 +/- 0.853 days |
| **Recovery** | 43.860% | 38.671% +/- 9.416% |

**NMR Spectrum**

o-CPPr diacid

p-CPPe diacid

o-CPPe prepolymer

p-CPPr prepolymer

p(o-CPPr:p-CPPe, 20:80)

**Reference**

1. Conix, A. Aromatic polyanhydrides, a new class of high melting fiber‐forming polymers. *J. Polym. Sci.* **29**, 343–353 (1958).

2. Shen, E., Pizsczek, R., Dziadul, B. & Narasimhan, B. Microphase separation in bioerodible copolymers for drug delivery. *Biomaterials* **22**, 201–210 (2001).

3. Berendsen, H. J. C., van der Spoel, D. & van Drunen, R. GROMACS: A message-passing parallel molecular dynamics implementation. *Computer Physics Communications* **91**, 43–56 (1995).

4. Wang, J., Wolf, R. M., Caldwell, J. W., Kollman, P. A. & Case, D. A. Development and testing of a general amber force field. *J Comput Chem* **25**, 1157–1174 (2004).

5. Sousa da Silva, A. W. & Vranken, W. F. ACPYPE - AnteChamber PYthon Parser interfacE. *BMC Res Notes* **5**, 367 (2012).

6. Neese, F. The ORCA program system. *WIREs Computational Molecular Science* **2**, 73–78 (2012).

7. Lee, C., Yang, W. & Parr, R. G. Development of the Colle-Salvetti correlation-energy formula into a functional of the electron density. *Phys. Rev. B* **37**, 785–789 (1988).

8. Hay, P. J. & Wadt, W. R. Ab initio effective core potentials for molecular calculations. Potentials for K to Au including the outermost core orbitals. *The Journal of Chemical Physics* **82**, 299–310 (1985).

9. Wang, J., Cieplak, P. & Kollman, P. A. How well does a restrained electrostatic potential (RESP) model perform in calculating conformational energies of organic and biological molecules? *Journal of Computational Chemistry* **21**, 1049–1074 (2000).

10. Darden, T., York, D. & Pedersen, L. Particle mesh Ewald: An *N* ⋅log( *N* ) method for Ewald sums in large systems. *The Journal of Chemical Physics* **98**, 10089–10092 (1993).

11. Ryckaert, J.-P., Ciccotti, G. & Berendsen, H. J. C. Numerical integration of the cartesian equations of motion of a system with constraints: molecular dynamics of *n*-alkanes. *Journal of Computational Physics* **23**, 327–341 (1977).
